# Coexisting Non‐Trivial Van der Waals Magnetic Orders Enable Field‐Free Spin‐Orbit Torque Magnetization Dynamics

**DOI:** 10.1002/adma.202502822

**Published:** 2025-07-01

**Authors:** Bing Zhao, Lakhan Bainsla, Soheil Ershadrad, Lunjie Zeng, Roselle Ngaloy, Peter Svedlindh, Eva Olsson, Biplab Sanyal, Saroj P. Dash

**Affiliations:** ^1^ Department of Microtechnology and Nanoscience Chalmers University of Technology Göteborg SE‐41296 Sweden; ^2^ Department of Physics Indian Institute of Technology Ropar Roopnagar 140001 India; ^3^ Department of Physics and Astronomy Uppsala University Box 516 Uppsala 75120 Sweden; ^4^ Department of Physics Chalmers University of Technology Göteborg 41296 Sweden; ^5^ Department of Materials Science and Engineering Uppsala University Uppsala SE‐751 03 Sweden; ^6^ Wallenberg Initiative Materials Science for Sustainability Department of Microtechnology and Nanoscience Chalmers University of Technology Göteborg SE‐41296 Sweden; ^7^ Graphene Center Chalmers University of Technology Göteborg SE‐41296 Sweden

**Keywords:** (Co_0.5_Fe_0.5_)_5‐x_GeTe_2_, 2D magnets, anti‐ferromagnet, exchange bias, ferromagnet, field‐free magnetization switching, room temperature, spin‐orbit torque

## Abstract

The discovery of van der Waals (vdW) magnetic materials exhibiting non‐trivial and tunable magnetic interactions can lead to exotic magnetic states that are not readily attainable with conventional materials. Such vdW magnets can provide a unique platform for studying new magnetic phenomena and realizing magnetization dynamics for energy‐efficient and non‐volatile spintronic memory and computing technologies. Here, the coexistence of ferromagnetic and antiferromagnetic orders in vdW magnet (Co_0.5_Fe_0.5_)_5‐x_GeTe_2_ (CFGT) above room temperature, inducing an intrinsic exchange bias and canted perpendicular magnetism is discovered. Such non‐trivial intrinsic magnetic order enables to realize energy‐efficient, magnetic field‐free, and deterministic spin‐orbit torque (SOT) switching of CFGT in heterostructure with Pt. These experiments, in conjunction with density functional theory and Monte Carlo simulations, demonstrate the coexistence of non‐trivial magnetic orders in CFGT, which enables field‐free SOT magnetization dynamics in spintronic devices.

## Graphical abstract

The coexistence of ferromagnetic and antiferromagnetic orders in van der Waals magnet above room temperature, inducing an intrinsic exchange bias and canted perpendicular magnetism is discovered. Such non‐trivial intrinsic magnetic order enables the realization of energy‐efficient and magnetic field‐free spin‐orbit torque memory devices.

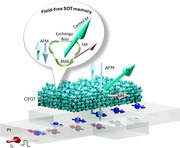

## 1 Introduction

Exploring new strategies to realize new non‐trivial magnetic orders is of great importance for fundamental magnetism, and also for the next generation of memory, logic, communication, and beyond von Neumann computing technologies.^[^ ^1^, ^2^ ^]^ Such non‐trivial magnetic states are required for efficient control of spin‐orbit torque (SOT) magnetization dynamics phenomena in spintronic devices for non‐volatile information and communication technologies.^[^ ^3^, ^4^, ^5^ ^]^ However, for energy‐efficient and field‐free SOT magnetization switching, the discovery of new materials and methods is needed for enhanced control over the device parameters.^[^ ^2^, ^6^, ^7^, ^8^, ^9^, ^10^, ^11^, ^12^, ^13^, ^14^, ^15^, ^16^, ^17^ ^]^ One of the strategies is to utilize heterostructures of ferromagnetic (FM) and antiferromagnetic (AFM) materials with exchange bias to realize field‐free SOT magnetization switching.^[^ ^7^, ^8^, ^18^, ^19^ ^]^ However, there are challenges in fabricating high‐quality FM/AFM interfaces with traditional multilayers, and the discovery of a single material with coexisting magnetic orders is necessary. In addition to conventional thin films, recently vdW FM/AFM heterostructures have been used to show field‐free SOT switching.^[^ ^19^ ^]^ However, achieving such phenomena in a single material will be a breakthrough for SOT memory technology.

Recently discovered two‐dimensional van der Waals (vdW) magnetic materials have significant potential for such unique non‐trivial magnetic states in a single material because of their low dimensionality, tunable magnetic anisotropy (MA), proximity‐induced interactions, and potential for voltage‐controlled magnetism and multi‐functionalities of SOT devices.^[^ ^2^, ^20^, ^21^, ^22^, ^23^, ^24^, ^25^, ^26^, ^27^ ^]^ Furthermore, it is possible to create unique, non‐trivial spin ordering by controlling the intra‐ and interlayer magnetic exchange coupling inherent to vdW materials, as well as the MA that depends on atomic position and stacking; however, this has not been explored.

**Figure 1:**
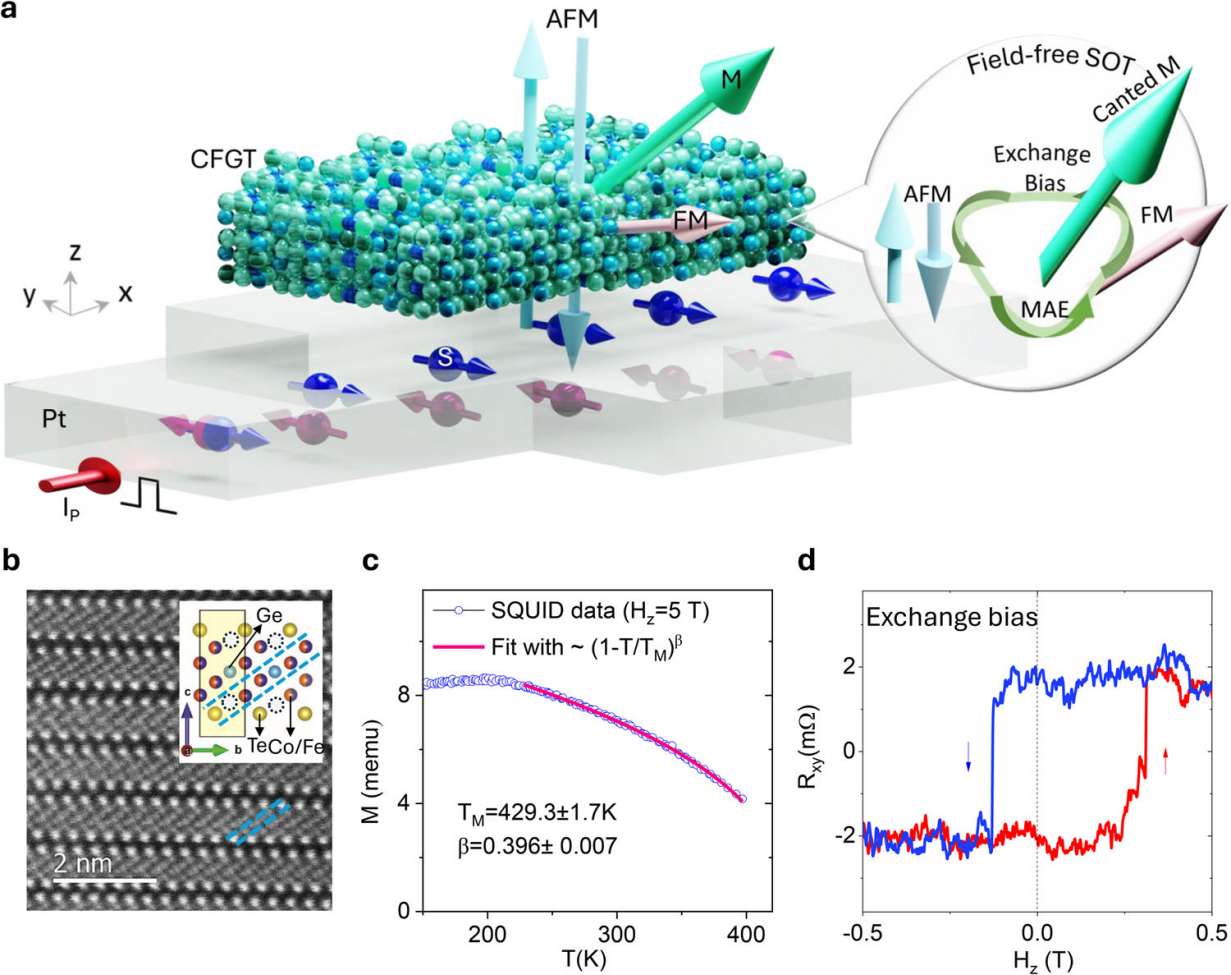
Coexistence of ferro‐ and antiferro‐magnetic orders in a single atomic stacking nanolayers of (Co_0.5_Fe_0.5_)_5‐x_GeTe_2_. a) Schematics of CFGT/Pt van der Waals heterostructure used for spin‐orbit torque (SOT) experiments. The ferro‐ (FM) and antiferro‐magnetic (AFM) orders in a single atomic stacking nanolayers, creating a net canted magnetization M with exchange bias interaction and magnetocrystalline anisotropy energy (MAE) (in the inset), which is useful for field‐free SOT switching. The charge current I_P_ applied to Pt generates a spin current J_s_ with spin polarizations S_y_ due to the spin Hall effect. b) Atomic resolution high angle annular dark field (HAADF) scanning transmission electron microscopy (STEM) images of CFGT. The inset is the atomic model projection along the bc‐plane. The dashed cycle represents Fe_1_ site vacancies. c) Temperature dependence of the magnetization M of bulk CFGT measured with a fixed out‐of‐plane magnetic field of 5T. The magnetic order temperature T_M_ is extracted with ~(1‐T/T_C(N)_)^β^ with critical factor β.^[^ ^28^, ^29^ ^]^ d) A representative AHE signal at 300 K with clear exchange bias effect (H_EB_ = +75 mT) after subtraction of a linear background at a ± 0.6T field range.

Here, we discover non‐trivial spin ordering with the coexistence of FM and AFM magnetic ordering in single vdW magnet (Co_0.5_Fe_0.5_)_5‐x_GeTe_2_ (CFGT), accompanied by a pronounced exchange bias effect and canted magnetization that persists above room temperature (see **Figure** **1**a), which is not readily attainable with conventional materials. By tuning the symmetry of the Fe_1_‐site vacancies, we successfully introduced a FM order in an AFM system. To understand the correlation between structural properties and the coexistence of FM and AFM orderings, we performed high‐throughput density functional theory (DFT) calculations and Monte Carlo simulations. This unique intrinsic canted magnetism of vdW magnet CFGT enables an energy‐efficient and deterministic field‐free spin‐orbit torque (SOT)‐induced magnetization switching when integrated with Pt in a heterostructure, owing to its high charge‐spin conversion efficiency, superior electrical conductance, and excellent spin transparency at the interface (see Figure 1a, inset).

## 2 Results and Discussion

### 2.1 Coexistence of Ferromagnetic and Antiferromagnetic Orders in CFGT at Room Temperature: Exchange Bias and Canted Magnetism

The motivation behind using the vdW magnet CFGT is its beyond room‐temperature magnetic orders and tunable magnetic properties depending on atomic positions, stacking, and intra‐ and interlayer magnetic interactions.^[^ ^26^, ^27^, ^30^ ^]^ Fe_5_GeTe_2_ exhibits an ABC stacking arrangement, characterized by a rhombohedral unit cell and belonging to the R $\bar{3}$ m space group.^[^ ^26^, ^27^ ^]^ By substituting around 50% of the Fe atoms with Co, crystals of CFGT alloy were synthesized.^[^ ^26^, ^27^, ^30^ ^]^ To characterize the atomic structure of CFGT, we performed high‐resolution scanning transmission electron microscopy (STEM) measurements as shown in Figure 1b (see details in Experimental and Note S1 and Figure S1, Supporting Information). We observe a uniform and homogeneous unique AA atomic crystal structure,^[^ ^30^ ^]^ with two different types of Fe_1_‐site atomic vacancy positions – an antisymmetric (type I) and a symmetric (type II) crystal structure with Fe_1_ deficiency sites (see more detailed analysis in Note S2, Figure S2, and Table S1, Supporting Information). Figure 1c shows the temperature‐dependent SQUID measurements as a function of a fixed out‐of‐plane field, confirming the magnetic behavior of CFGT with a magnetic order temperature T_M_ = 429.3 ± 1.7 K.

**Figure 2:**
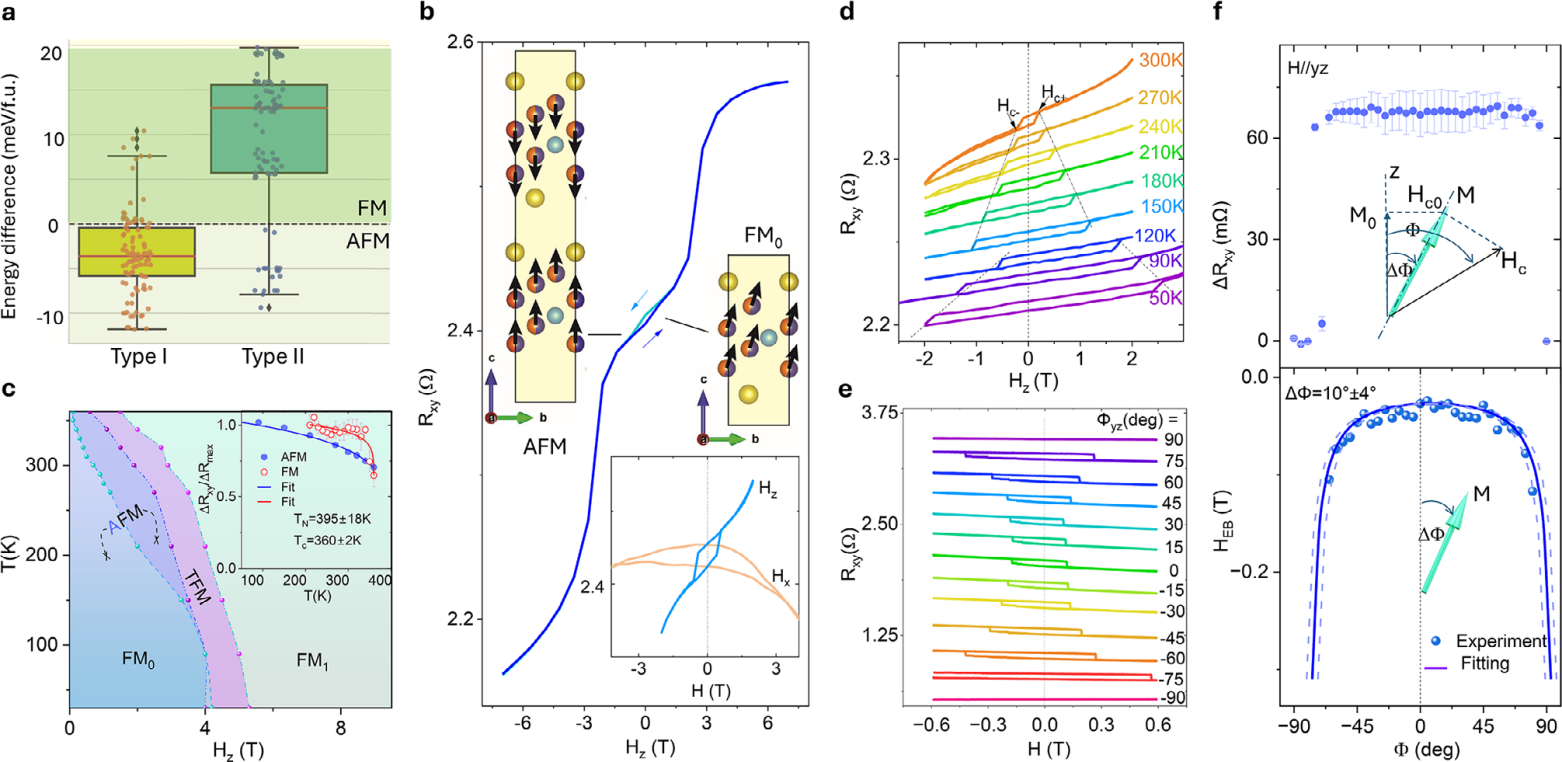
Origin of the (A)FM magnetic orders and the canted magnetization M in (Co_0.5_Fe_0.5_)_5‐x_GeTe_2_ nanolayers. a) Boxplots of the magnetic ground state distribution of AFM (FM) orders in CFGT, where each dot represents a distinct Co distribution. The orange lines are the statistically averaged results. b) Transversal Hall resistance (AHE) R_xy_ = V_xy_/I of a CFGT nanolayers as a function of the out‐of‐plane field (H_z_) at 300 K in the field range of ±8 Tesla. The blue/green arrows show the AFM and FM magnetic spin configurations. The schematics show the magnetic states near 0 T. Inset: AHE signals at 300 K in a smaller field range with H_z(x)_. c) Phase diagram of the CFGT nanolayers as a function of H_z_ and T. Inset shows the temperature evolution of the normalized AFM and FM components. The Curie (Néel) temperature T_C(N)_ is extracted by fitting with ≈(1‐T/T_C(N)_)^β^. d) AHE signals R_xy_ with out‐of‐plane field H_z_ at different temperatures in the low field range. H_c±_ are the coercive fields in positive/negative directions. The dashed lines are guide to the eye. e) AHE signals R_xy_ with magnetic field sweep at different yz‐plane angles Φ measured at 300 K. f) The magnitude of the AHE signal ΔR_xy_ and exchange bias field H_EB_ = (H_c+_+H_c‐_)/2 as a function of the yz‐plane angle Φ. The purple curve in the bottom panel is a fit with uncertainty represented by dashed curves. The insets show the schematics of the magnetization M at a canting angle ∆Φ and its relationship with the external field H_c_.

To investigate the magnetic properties of thin CFGT nanolayers, magnetotransport measurements using the anomalous Hall effect (AHE) were conducted on nanofabricated Hall‐bar devices (see Experimental section). Figure 1d shows AHE data (Hall resistance R_xy_ as a function of the out‐of‐plane magnetic field H_z_) at 300 K, revealing a clear hysteresis loop that confirms the presence of out‐of‐plane ferromagnetism in CFGT with finite remanence and coercivity at room temperature. More interestingly, a strong exchange bias effect is observed, which is manifested as a horizontal shift in the magnetic hysteresis loop due to the coexistence of intrinsic FM and AFM ordering in CFGT. Our density functional theory (DFT) calculations also show the coexistence of FM and AFM magnetic states. The boxplots in **Figure** **2**a illustrate these structural dependencies of calculated magnetic ground states, where each dot represents a distinct Co distribution. The y‐axis shows the energy difference between FM and AFM ordering for each configuration. Negative values indicate a preference for AFM order, while positive values correspond to FM order in the unit cell. We have calculated hundreds of different possible Co‐doping cases for Type I (II) structures. The statistically averaged results suggest that the Type I (II) structures with all possible Co doping show the AFM (FM) ground magnetic state, respectively (see details in Note S3 and Table S2, Supporting Information). The detailed AHE measurements of CFGT nanolayers in both low and high field ranges confirmed the coexistence of FM and AFM states at room temperature below the spin‐flip transition, as shown in Figure 2b. From both experiments and theoretical analysis, we observe that our CFGT sample is primarily in the AFM state, and we introduced a coexisting FM state due to the presence of symmetric Fe_1_ vacancy sites. We further evaluated the temperature evolution of the FM (AFM) magnetic orders (Figure S3, Supporting Information). The extracted magnetic phase diagram for the CFGT nanolayers as a function of H_z_ and temperature T is shown in Figure 2c. In a small field range, the FM_0_ and AFM orders coexist. A transitional magnetic order (TFM) appears when the field is large enough to re‐align the AFM components. Finally, both AFM and FM_0_ magnetic orders are aligned with the external field direction to form the ferromagnetic FM_1_ order. Based on the evolution of magnitude of AFM(FM) components in the AHE signals (Figure 2c, inset), we extract the Curie (Néel) temperature T_C_ = 360 ± 2 K (T_N_ = 395 ± 15 K) of CFGT nanolayers. Furthermore, Figure 2d illustrate a more detailed temperature evolution of the AHE signal, where apparent hysteresis is observed in a smaller magnetic field sweep, i.e., with the ferromagnetic FM_0_ order. At lower temperatures, the coercivity H_c±_ increases, and the exchange bias effect becomes more prominent below 150 K. To be noted, the Curie temperature (T_c_) for the ferromagnetic orders in CFGT is around 429 K for bulk samples and 360 K for nanolayers, among the highest T_c_ among all the known 2D vdW magnets (like Fe_3_GaTe_2_;^[^ ^31^ ^]^ Fe_5_GeTe_2_;^[^ ^32^ ^]^ Fe_3_GeTe_2_;^[^ ^33^ ^]^ CrTe_2_;^[^ ^34^ ^]^ CrI_3_;^[^ ^35^ ^]^ etc. see Figure S12b, Supporting Information).

Due to the coexistence of both FM and AFM properties in CFGT nanolayers, there is a possibility that its MA may be significantly impacted. To examine this, we conducted an angle‐dependence study of the AHE on the CFGT Hall‐bar device at room temperature (Figure 2e). The AHE signal with exchange bias effect can be observed at all angles, except for the almost in‐plane case Φ ≈ ±90 deg where the signal vanishes due to no effective field to switch the M. As shown in Figure 2e, the magnitude of the AHE signal remains almost constant at all field sweep angles Φ, indicating a strong MA and minimal rotation of the magnetization towards the field direction in the range of ±600 mT. The extracted exchange bias field H_EB_ at different angles is presented in Figure 2f (bottom panel). We consider H_c0_ to be the intrinsic coercive field required to switch M with a magnetic field aligned with the easy axis of the magnetization. The projection of the nominal coercive field H_c_(Φ) at field sweep angle Φ on the easy axis must be equal to H_c0_, that is, H_c0_ = H_c_ cos(Φ‐*∆*Φ), where *∆*Φ defines the canted angle of the magnetization easy axis (Figure 2e, insets schematics). Then the relation H_EB_(Φ) = H_EB0_/cos(Φ‐*∆*Φ) with the field rotation angle Φ can be formulated to fit the angle‐dependent H_EB_ (see detailed analysis in Note S4, Supporting Information), yielding a magnetization canting angle ∆Φ of ≈10 degrees in CFGT (see also similar results from another device in Figure S4, Supporting Information). The canted magnetism is further confirmed by our DFT calculations and Monte Carlo simulations, which suggest that the intrinsic exchange bias and magnetocrystalline anisotropy energy of CFGT are the origin of canted magnetization (see detailed analysis in Note S5, Figures S5 and S6, Supporting Information).

### 2.2 Field‐Free Spin‐Orbit Torque Switching of CFGT/Pt Heterostructure Devices at Room Temperature

**Figure 3:**
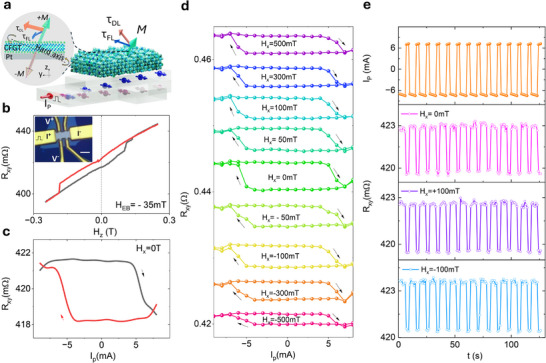
Field‐free spin‐orbit torque magnetization switching in CFGT/Pt heterostructure at room temperature. a) Schematics of CFGT/Pt van der Waals heterostructure used for pulsed current I_P_‐induced spin‐orbit torque (SOT) experiments. Inset shows the mechanism of the field‐free SOT switching of CFGT. b) AHE signals with an exchange field H_EB_ = ‐35 mT. The inset shows the optical microscope image of the CFGT/Pt heterostructure Hall‐bar device. The scale bar is 2 µm. c, d) Pulsed write current I_p_ induced transverse Hall signal R_xy_ change due to SOT‐induced magnetic switching without/with external in‐plane magnetic field H_x_. e) Time dependence of the pulse current I_p_ and the corresponding measured AHE signal R_xy_ with in‐plane fields H_x_ = 0, and ±100 mT. The pulse current dwell time is 1 ms and 3s waiting time before the reading process. All the measurements here were performed in Dev 1.

SOT phenomenon, which utilizes the spin‐orbit interaction in materials and their hybrid structures with magnets, has emerged as an efficient method to control the magnetization in spintronic devices.^[^ ^3^, ^4^, ^5^ ^]^ For the success of SOT‐driven magnetization dynamics,^[^ ^3^, ^4^, ^5^ ^]^ it is required to enhance the SOT efficiency, achieve magnetic field‐free magnetization switching, and discover new materials and methods to control the device parameters^[^ ^2^, ^6^, ^7^, ^8^, ^9^, ^10^, ^11^, ^12^, ^13^, ^14^, ^15^, ^16^ ^]^ (**Figure** **3**a). To investigate the SOT‐induced magnetization switching and dynamics in CFGT samples with intrinsic exchange bias and canted magnetization effects, CFGT/Pt heterostructure Hall bar devices were nanofabricated as shown in Figure 3b inset (see details in the Experimental section). For an effective SOT magnetization switching, it is essential to have a large charge‐spin conversion efficiency in Pt and high‐quality CFGT/Pt interfaces for efficient transmission of spin current. In the SOT‐induced magnetization switching experiment, a series of DC pulse currents I_p_ applied along the x‐direction through the spin‐orbit material (Pt), generates a spin current along the z‐axis with spin polarization *s_y_* along the y‐axis due to the spin Hall effect. The resulting spin current exerts SOTs in CFGT (field‐like *τ_FL_* and damping‐like *τ_DL_* torques), enabling the switching of magnetization *M* direction. Specifically, the field‐like torque *τ_FL_* ≈ *M*×*s_y_*, precesses *M* about the exchange field created by spin polarization, while the damping‐like torque *τ_DL_* ≈ *M*×(*M*×*s_y_*) rotates the magnetization *M* toward the direction of spin polarization *s_y_*. Typically, damping‐like torque determines the switching of the magnetization.^[^ ^36^ ^]^

The AHE signal of the CFGT/Pt Hall device shows an out‐of‐plane field magnetization and the difference of the switching field for both field sweep directions suggest a strong exchange bias (H_EB_ = ‐35 mT) in CFGT (Figure 3b). As shown in Figure 3c, we observed the SOT‐induced magnetization switching using a pulsed write current I_p_ with pulse time 1 ms, followed by a small DC read current (I_r_≈50‐500 µA) to probe the magnetization state R_xy_ = V_xy_/I_r_. As the signal R_xy_ is proportional to the out‐of‐plane magnetization *M_z_*,^[^ ^41^ ^]^ the SOT R_xy_ signal shows current‐induced magnetization change between *+M* and *‐M*. The magnitude change of R_xy_ in the SOT signal is almost the same as the AHE signal with field sweep, suggesting a full FM magnetization switch. Interestingly, we observe a deterministic SOT switching of CFGT at H_x_ = 0 T. This is in contrast to the conventional requirement of an in‐plane magnetic field H_x_ to break the geometrical symmetry for a deterministic switching of the magnetization with a perpendicular magnetic anisotropy (PMA).^[^ ^37^ ^]^ To verify the origin, we performed SOT measurements with different in‐plane magnetic fields H_x_ (Figure 3d). The H_x_ field does not affect the deterministic SOT switching, and the magnetization switching direction (chirality) remains the same in this field range of ±500 mT, suggesting a robust deterministic switching against the external magnetic field due to the strong exchange bias effects and the canted magnetization of CFGT. The time‐dependent switching experiments with different H_x_ were carried out (Figure 3e), further proving the reproducibility of the deterministic switching (also see reproducibility of SOT switching experiment Figures S7 and S8 (Supporting Information), discussion about the origin of the field‐free SOT‐induced magnetization switching in Note S6 (Supporting Information), and critical switching current density in Table S3, Supporting Information).

Conventionally, chirality is determined by the external field, which favors opposite switching directions for the positive and negative in‐plane magnetic fields.^[^ ^37^ ^]^ If no external field is applied, the magnetization would be pulled to the in‐plane orientation and broken into multiple domains, instead of the deterministic switching. However, in the canted magnetization scenario of the CFGT sample (Figure 3a, inset), the symmetry is broken as the easy axis of the magnetization *M* is canted at an angle ΔΦ with respect to the normal direction. The damping‐like torque *τ*_DL_ can rotate the *M* anticlockwise over the hard axis towards another easy axis, while the other rotation direction is not possible. This also explains the unchanged chirality of SOT switching with the external field direction, which is similar to that observed in other canted magnets.^[^ ^38^ ^]^ Thermal fluctuations induced by pulse current can randomize the magnetic domain of CFGT and make switching behavior unstable.^[^ ^39^ ^]^ This is not the case in our CFGT/Pt SOT device, although the reduction of the SOT signal at a larger current due to thermal fluctuations cannot be ruled out.^[^ ^40^ ^]^ We can also rule out thermally assisted switching if the device reaches above T_c_, as this can result in non‐deterministic switching due to the formation of multiple domains.^[^ ^41^, ^42^ ^]^ Therefore, the observed deterministic and field‐free switching of CFGT can be attributed to the dominant SOT contribution and the canted magnetization of CFGT.

### 2.3 Estimation of Spin‐Orbit Torque Magnetization Dynamics using 2nd Harmonic Hall Measurement in CFGT/Pt Heterostructure Devices

**Figure 4:**
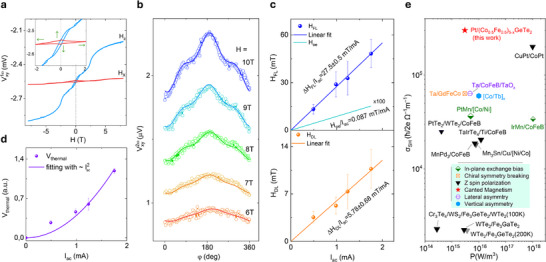
Harmonic Hall measurements of the CFGT/Pt Hall‐bar device at room temperature. a) 1st harmonic V_xy_^1ω^ signals as a function of the out‐of‐plane field H_z_ and in‐plane field H_x_ sweeps. The inset is the zoom‐in around 0 T. The green arrows show the FM components. b) In‐plane angle φ dependence of the 2nd Harmonic signal V_xy_^2ω^ at I_ac_ = 1.75 mA with in‐plane field H. c, d) Extracted current dependence of the field‐like effective field H_FL_, Oersted field H_Oe_, damping‐like effective field H_DL,_ and thermal contribution. All the harmonic measurements were performed with ω = 213.14 Hz at 300 K in Dev 2. e) Benchmarking our Pt‐CFGT results with the state‐of‐the‐art field‐free magnetization switching devices reported in the literature such as PtMn/[Co/Ni],^[^ ^7^ ^]^ IrMn/CoFeB,^[^ ^8^ ^]^ Ta/GaFeCo,^[^ ^43^ ^]^ CuPt/CoPt,^[^ ^44^ ^]^ TaIrTe_4/_Ti/CoFeB,^[^ ^16^ ^]^ PtTe_2_/WTe_2_/CoFeB,^[^ ^45^ ^]^ MnPd_3_/CoFeB,^[^ ^46^ ^]^ Mn_3_Sn/Cu/[Ni/Co],^[^ ^47^ ^]^ WTe_2_/Fe_3_GaTe_2_,^[^ ^48^ ^]^ WTe_2_/Fe_3_GeTe_2_,^[^ ^6^ ^]^ Cr_3_Te_4_/WS_2_/Fe_3_GeTe_2_/WTe_2_,^[^ ^49^ ^]^ Ta/CoFeB/TaOx,^[^ ^9^ ^]^ [Co/Tb]_x_.^[^ ^50^ ^]^ Pt‐based field assistant SOT devices: Pt/Fe_3_GeTe_2_,^[^ ^39^, ^51^ ^]^ Pt/Fe_3_GaTe_2_.^[^ ^52^ ^]^ Spin Hall conductivity σ_SH_ as a function of the dissipation power density for magnetization switching P = (J_sw_^2^/σ_c_), where J_sw_ is the switching current density and σ_c_ is the conductance of the spin source material. Different field‐free mechanisms are indicated with the symbols.

Harmonic Hall measurement is a powerful tool for studying different SOT contributions and effective spin‐orbit fields in magnetic heterostructures.^[^ ^53^ ^]^ Application of an AC current I_ac_ = I_0_sin*ω*t with frequency *ω* modulates the SOT amplitude and induces small oscillations of *M* about its equilibrium direction.^[^ ^44^ ^]^ Such oscillations generate a 2nd harmonic contribution to the Hall voltage^[^ ^54^ ^]^ (V_xy_≈V_0_+ $V_{xy}^{1\omega }$ sin*ω*t+ $V_{xy}^{2\omega }$ cos2*ω*t), where V_xy_ depends on M_z_ through the AHE and the product of *M_x_* x *M_y_* through the planar Hall effect (PHE). The 1st harmonic term $\;V_{xy}^{1\omega }$ relates to the equilibrium direction of the magnetization and is independent of the modulated SOT, which was measured as an in‐phase (Ø_lockin_ = 0^о^) signal. In the 1st harmonic Hall signals $V_{xy}^{1\omega }\;$ versus H_z_ field sweep (**Figure** **4**a), we observed the expected AFM order transition and FM hysteresis in high and low field ranges, respectively, whereas the H_x_ field sweep measurement shows the FM magnetization component being pulled to the in‐plane direction at around 2 T (i.e., magnetic anisotropy field, H_k_), without any clear indication of the AFM transition up to 7 T.

The out‐of‐phase (Ø_lockin_ = 90^о^) 2nd harmonic term $V_{xy}^{2\omega }$ measures the response of the magnetization to the current‐induced spin‐orbit fields. The current‐induced effective field H_eff_ can be decomposed into two components: the field‐like (H_FL_) and damping‐like (H_DL_) spin‐orbit fields, which can be extracted by the following equation,^[^ ^53^ ^]^

(1) $$\begin{array}{ccc} V_{xy}^{2\omega } & & =-\left(V_{AHE}\frac{H_{DL}}{H-H_{k}}+V_{thermal}\right)\;cos\varphi +2V_{PHE}\frac{H_{FL}+H_{oe}}{H}\\ & & \left(2cos^{3}\varphi -cos\varphi \right) \end{array}$$

where H is the applied external field; φ is the angle between the applied current and the field. V_AHE_ and V_PHE_ are the AHE voltage and planar Hall (PHE) voltage, respectively. V_thermal_ is the thermal‐related contribution, like anomalous Nernst effect and spin Seebeck effect, etc. By adopting the simplified model,^[^ ^55^ ^]^ the current induced Oersted field H_oe_ = I_Pt_/(2 W) is also calculated, where W is the width of the Hall device. Therefore, the 2nd harmonic Hall voltage signal $V_{xy}^{2\omega }\;$ as a function of an in‐plane rotation φ with fixed magnetic field H can be measured to extract the contributions from H_DL_, H_FL,_ and the current‐induced thermal effect (Figure 4b). Furthermore, the current dependence of the contributions from H_DL_, H_FL,_ and the thermal effects are plotted in Figure 4c,d. We extracted ∆H_DL_/J_ac_ = 4.2 mT per MA/cm^2^ with an effective saturation magnetization µ_0_M_s_ ≈ 2.1 emu cm^−3^ and ∆H_FL_/J_ac_ = 19.7 mT per MA cm^−2^ (see more details on the harmonic Hall measurement methods in Note S7, Figures S10 and S11, Supporting Information, and the summary of the key SOT device parameters in Table S3, Supporting Information). The µ_0_M_s_ is smaller than the value of pure FM magnetic order with the AA’ crystal structure.^[^ ^56^ ^]^ This suggests that the AFM order does not contribute much to the effective saturation magnetization even at large in‐plane fields but coexists with the FM order, which agrees with the 1st harmonic result with H_x_ sweep.

The current‐induced Oersted field is negligible and cannot be the origin of the field‐free SOT‐induced magnetization switching, while thermal contribution to the harmonic Hall voltage signal $\;V_{xy,\;thermal}\propto I^{2}$, increasing quadratically with the current, is proportional to *M_x_*. Geometrically, $V_{xy,\;thermal}$ ≈ *M_x_* × *∇T_z_*, where *∇T_z_* is due to the different thermal conductivity of CFGT, Pt, SiO_2_/Si substrate, and air. Utilizing both SOT‐induced magnetic switching and 2nd harmonic Hall measurements, we can conclude that the canted magnetization in CFGT can be efficiently controlled with a switching current density of J_sw_≈8 × 10^6^ A cm^−2^ at 300 K, which is lower than the reported values for vdW magnets.^[^ ^39^, ^51^, ^52^ ^]^ Moreover, as benchmarked against the state‐of‐the‐art representative field‐free SOT devices (Figure 4e), including devices with lateral asymmetry (mirror symmetry breaking), chiral symmetry breaking, in‐plane exchange bias, vertical asymmetry, out‐of‐plane spin polarization S_z_, Our Pt/CFGT devices show a very low operating power of ≈10^15 ^W m^−3^ for the deterministic magnetization switching. Such highly efficient SOT‐induced magnetization switching can be mainly due to the large spin Hall angle, high electrical conductance of Pt, and excellent spin transparency of the CFGT/Pt interface. Moreover, CFGT's lower conductivity compared to traditional 3D FMs such as Co and CoFeB enables a larger current to flow through the Pt, thereby enhancing energy efficiency. Noticeably, the *σ_SH_* (≈ 3 × 10^5^ ħ/ 2e Ω^−1^m^−1^) of our device is comparable with other Pt‐based field assistant SOT devices; however, it requires less power due to the small net magnetization µ_0_M_s_ of CFGT (Figure 4e; also see state‐of‐the‐art vdW magnet‐based SOT devices in Note S8 and Figure S12a, Supporting Information). Alternatively, spin‐orbit materials with lower crystal symmetry, such as WTe_2_ and TaIrTe_4_, are used to deterministically switch the magnetization without an external field due to an out‐of‐plane SOT component.^[^ ^6^, ^11^, ^15^, ^16^, ^57^ ^]^ However, the unconventional SOT efficiency of WTe_2_ and TaIrTe_4_ from S_z_ spin component is still much smaller than that from *S*_y_ component of Pt.^[^ ^6^, ^15^, ^16^, ^57^ ^]^ Therefore, the canted magnetism of CFGT enables a more energy‐efficient and deterministic field‐free SOT‐induced magnetization switching in a heterostructure with Pt.

Importantly, for SOT memory devices, one of the traditional strategies is to utilize bilayers of FM/AFM thin films to achieve exchange bias and canted magnetism to realize field‐free SOT magnetization switching, where AFM is just used as a pinned/exchange bias layer.^[^ ^7^, ^8^, ^19^, ^58^ ^]^ However, our finding of the intrinsic coexistence of FM and AFM orders with exchange bias and canted magnetism in single magnetic materials CFGT enables us to demonstrate an energy‐efficient and deterministic field‐free SOT‐induced magnetization switching of the vdW magnet CFGT. Furthermore, the low net magnetization of CFGT can also be useful for different device applications, for example, as shown for topological antiferromagnet Mn_3_Sn^[^ ^59^ ^]^ and compensated ferrimagnets (with almost zero magnetization) for ultra‐fast and energy‐efficient switching.^[^ ^50^, ^60^, ^61^, ^62^ ^]^

## 3 Conclusion and Perspective

We observe the coexisting FM and AFM properties in vdW magnet CFGT much above room temperature, with intrinsic exchange bias and canted perpendicular ferromagnetism, which is not possible to attain in conventional systems. We understand the correlation between structural properties and coexisting magnetic orders using high‐throughput DFT calculations utilizing data‐driven analysis. It shows that such unique magnetic orders can be controlled by the positions of vacancies in the crystal and their symmetries, offering a new strategy for synthesizing novel vdW magnetic materials. Such intrinsic coexistence of non‐trivial magnetic orders enables the demonstration of an energy‐efficient, field‐free, and deterministic switching of the vdW magnet CFGT in heterostructure with conventional material Pt with a large spin Hall conductivity. The detailed investigation of magnetic properties of CFGT and magnetization dynamics using SOT switching and 2nd harmonic Hall measurements of CFGT/Pt heterostructures show their relationship with the observed field‐free SOT behavior. By demonstrating the feasibility of deterministic SOT‐induced magnetization switching in a non‐trivial vdW magnet at room temperature without the application of an external magnetic field, our work lays the foundation for the development of efficient spintronic devices based on vdW magnets. Moreover, intrinsically CFGT hosts both FM and AFM magnetic orders with a canted out‐of‐plane magnetism, offering the flexibility to combine other materials with even larger charge‐spin conversion efficiency to enhance the device's performance.

## 4 Experimental Section

### Device Fabrication

Single crystals of (Fe_0.5_Co_0.5_)_5_GeTe_2_ (CFGT) were grown by chemical vapor transfer (CVT) method by HqGraphene. The AHE measurements were measured in Hall‐bar devices of CFGT (15‐60 nm) on SiO_2_/Si substrate. The CFGT crystal was exfoliated onto the SiO_2_/Si wafer, followed by electron‐beam lithography (EBL) and Ar plasma etching to pattern in a Hall‐bar geometry, and the Au/Ti contacts were deposited 2nd EBL step and lift‐off technique. The CFGT/Pt heterostructures were prepared by exfoliating thin CFGT crystals (15‐60 nm) inside an N_2_ glovebox onto pre‐deposited Pt(10 nm)/Ti(2 nm) layers on a SiO_2_/Si substrate. The Pt layer was prepared by electron‐beam evaporation onto a Si/SiO_2_ substrate with a Ti seed layer. After exfoliation of CFGT, the samples were immediately transferred to an electron‐beam evaporation system to deposit a 2 nm Al layer, forming an AlO_x_ capping layer. The Hall bar and contacts were patterned using EBL and Ar ion beam etching.

### Electrical Measurements

Low temperature and high magnetic field measurements of CFGT Hall bar devices were performed in the Quantum Design cryogen‐free PPMS DynaCool system with bias current in the range of 50–500 µA in the temperature range of 10–360 K and magnetic field up to 13 Tesla (T).

### SOT Switching and Harmonic Measurements

SOT switching and Harmonic measurements in a low magnetic field range were carried out in a vacuum cryostat with a magnetic field up to 0.7 T for CFGT/Pt devices. The electronic measurements were performed using a Keithley 6221 current source, and a nanovoltmeter 2182A. To monitor the longitudinal and transverse Hall resistances, Keithley 2182A nanovoltmeters were utilized. For SOT‐induced magnetization switching measurements, Keithley 2182A nanovoltmeters were used to monitor the response of the Hall resistances, while a Keithley 6221 AC source was utilized with a pulse current of 1 millisecond (ms) through the device, followed by a DC read current. The harmonic measurement was performed using Lockin SR830 to measure in‐phase 1st and out‐of‐phase 2nd harmonic voltages, respectively. The 2^nd^ harmonic measurements in the high magnetic field range were carried out in the Quantum Design cryogen‐free PPMS DynaCool system with an external electronic connection to Lockin SR830 to measure the 1st and 2nd harmonic voltages.

### SQUID Measurements

A Quantum Design superconducting quantum interference device (SQUID) was used to measure the static magnetic properties of bulk CFGT crystals. We measured the magnetization as a function of the out‐of‐plane (H∥z) magnetic field. The bulk CFGT crystal was glued on a Si substrate to properly align the sample in the magnetic field during the measurements.

### STEM Measurements

Scanning transmission electron microscopy (STEM) measurements were carried out using a JEOL monochromatic ARM200F transmission electron microscope (TEM), which is equipped with a Schottky field emission gun, a double‐Wien monochromator, a probe Cs corrector and an image Cs corrector, as well as high angle annular dark field (HAADF) detectors for STEM imaging, a double silicon drift detector (SDD) for energy dispersive X‐ray spectroscopy (EDXS), and a Gatan image filter (GIF) Continuum. The microscope was operated at 200 kV for HAADF STEM imaging. The beam convergence half‐angle and inner collection half‐angle for HAADF STEM are ≈ 27 mrad and ≈ 55 mrad, respectively. Specimens for STEM measurements were prepared by using a FEI Versa 3D focus ion beam–scanning electron microscope (FIB‐SEM). After depositing a protection layer containing Pt and C using first the electron beam and then the Ga ion beam in the FIB‐SEM, a lamella of the material was cut out using an ion beam at 30 kV and 1 nA. After transferring the lamella to a Cu TEM grid, the lamella was gradually thinned down by the ion beam. The thinning process was performed first at 30 kV and with a gradually decreasing beam current from 1 nA to 100 pA. Then, the gentle polishing of the specimen was carried out with an ion beam energy of 5 kV and 2 kV to minimize the ion beam effect.

### Statistical Analysis

Statistical analysis was conducted using Python 3.12 with the libraries numpy, scipy.stats, and matplotlib. Outlier detection was performed using the interquartile range (IQR) method, and all data points—including identified outliers—were retained and visualized in the final boxplots to ensure full transparency. No transformation or normalization was applied. Data are presented using boxplots with overlaid individual data points. Each box displays the interquartile range (IQR; 25th to 75th percentile) with the median marked as a horizontal line. Whiskers extend to the most extreme values within 1.5×IQR from the lower and upper quartiles. Points beyond the whiskers are plotted as outliers. Sample sizes were n1 = 100 for Type I (AFM) and n2 = 100 for Type II (FM). To assess the statistical significance in energy differences between the two compositions, the non‐parametric Mann–Whitney U test was employed due to violations of normality. A statistically significant difference was found between the groups (U = 8604.0, p = 1.31 × 10^−18^), with the significance level set at α = 0.05.

## Conflict of Interest

The authors declare no conflict of interest.

## Author Contributions

B.Z. and S.P.D. conceived the idea and designed the experiments. B.Z. fabricated and characterized the devices with some support from L.B. and R.N. S.E. and B.S. performed the theoretical calculations and data analysis. L.Z. and E.O. performed STEM characterization and data analysis. P.S. performed SQUID magnetization measurements and data analysis. B.Z., S.E., B.S, and S.P.D. analyzed and interpreted the data, compiled the figures, and wrote the manuscript with feedback from all co‐authors. S.P.D. supervised and managed the research project.

## Supporting information



Supporting Information

## Data Availability

The data that support the findings of this study are available from the corresponding author upon reasonable request.
