# Supplementary material for: Coexisting Non‐Trivial Van der Waals Magnetic Orders Enable Field‐Free Spin‐Orbit Torque Magnetization Dynamics
Source: Adv Mater. 2025 Jul 1;37(37):2502822. doi: 10.1002/adma.202502822 (PMC12447057; doi:10.1002/adma.202502822)
Supplement: Supplementary file 1 — Supporting Information [file ADMA-37-2502822-s001.pdf]

ADVANCED MATERIALS

Supporting Information

for *Adv. Mater.*, DOI 10.1002/adma.202502822

Coexisting Non-Trivial Van der Waals Magnetic Orders Enable Field-Free Spin-Orbit Torque Magnetization Dynamics

Bing Zhao, Lakhan Bainsla, Soheil Ershadrad, Lunjie Zeng, Roselle Ngaloy, Peter Svedlindh, Eva Olsson, Biplab Sanyal* and Saroj P. Dash**

Supplementary information

Coexistence of non-trivial van der Waals magnetic orders enable field-free spin-orbit torque switching at room temperature

Bing Zhao[1*], Lakhan Bainsla[1,2], Soheil Ershadrad[3], Lunjie Zeng[4], Roselle Ngaloy[1], Peter Svedlindh[5],

Eva Olsson[4], Biplab Sanyal[3*], Saroj P. Dash[1,6,7*]

[1]Department of Microtechnology and Nanoscience, Chalmers University of Technology, SE-41296, Göteborg, Sweden.
[2]Department of Physics, Indian Institute of Technology Ropar, Roopnagar 140001, India.
[3]Department of Physics and Astronomy, Uppsala University, Box-516, 75120 Uppsala, Sweden.
[4]Department of Physics, Chalmers University of Technology, 41296, Göteborg, Sweden.
[5]Department of Materials Science and Engineering, Uppsala University, Uppsala, SE-751 03 Sweden.
[6]Wallenberg Initiative Materials Science for Sustainability, Department of Microtechnology and Nanoscience, Chalmers University of Technology, SE-41296, Göteborg, Sweden.
[7]Graphene Center, Chalmers University of Technology, Göteborg, SE-41296 Sweden.

Content:

Supplementary Note 1. STEM characterization of CFGT
Supplementary Note 2. Unique AA-stacked structures of CFGT
Supplementary Note 3. DFT calculation results and Computational methods
Supplementary Note 4. Extraction of the magnetization canted angle ΔΦ in CFGT
Supplementary Note 5. The origin of the canted magnetization
Supplementary Note 6. Origin of the field-free SOT-induced magnetization switching
Supplementary Note 7. Harmonic Hall measurements for SOT quantification
Supplementary Note 8. State of the art of van der Waals magnet-based SOT devices
Supplementary Note 9. Possibility of exchange bias effect caused by oxidation on the surface layer

Supplementary Note 1. STEM characterization of CFGT

To characterize the atomic structure of $(Co_{0.5}Fe_{0.5})_5GeTe_2$ (CFGT), we prepared two TEM specimens along two different directions [110] and [1-10] from the bulk crystals (Fig. S1a). The HAADF STEM results on two samples are presented (Fig. S1b and Fig. S1c), with the corresponding structure models[1], respectively. The atomic structure in the STEM images is identical to the model. The atomic profile along the TeFe(Co)GeTe chain (indicated with dashed lines) shows the zig stacking. There are comparable 6 or 5 (FeCoGe) atoms in the atomic chain between a pair of Te atoms. A large-scale STEM image shows that CFGT is a homogeneous system (Fig. S1d). According to the STEM results (Fig. S2a and Fig. S2d) and the extracted atomic profiles (Fig. S2b and Fig. S2e) along the TeFe(Co)GeTe atom chain (indicated by the orange and blue dashed lines in Fig. S2a and Fig. S2d), it can be observed that in type I, one of the Fe_1-sites is vacant, which we define as a Fe_1 -site vacancy, while other atomic sites are occupied by TeFe(Co)GeTe atoms. Therefore, type I exhibits an

asymmetric Fe distribution with respect to the Ge atom (Fig. S2b). In contrast, type II has a pair of symmetric Fe_1-site vacancies (Fig. S2e).

We then plot the corresponding schematics to visualize and support the symmetric and asymmetric Fe coordination. As shown in Fig. S2c for type I, the yellow highlighted area illustrates the TeFe(Co)GeTe atoms chain, featuring an asymmetric distribution of the Fe_1-site vacancy with respect to the Ge atom. In contrast, for type II, a pair of symmetric Fe_1-site vacancies are present in the TeFe(Co)Ge atom chain (highlighted in the blue area) in Fig. S2f.

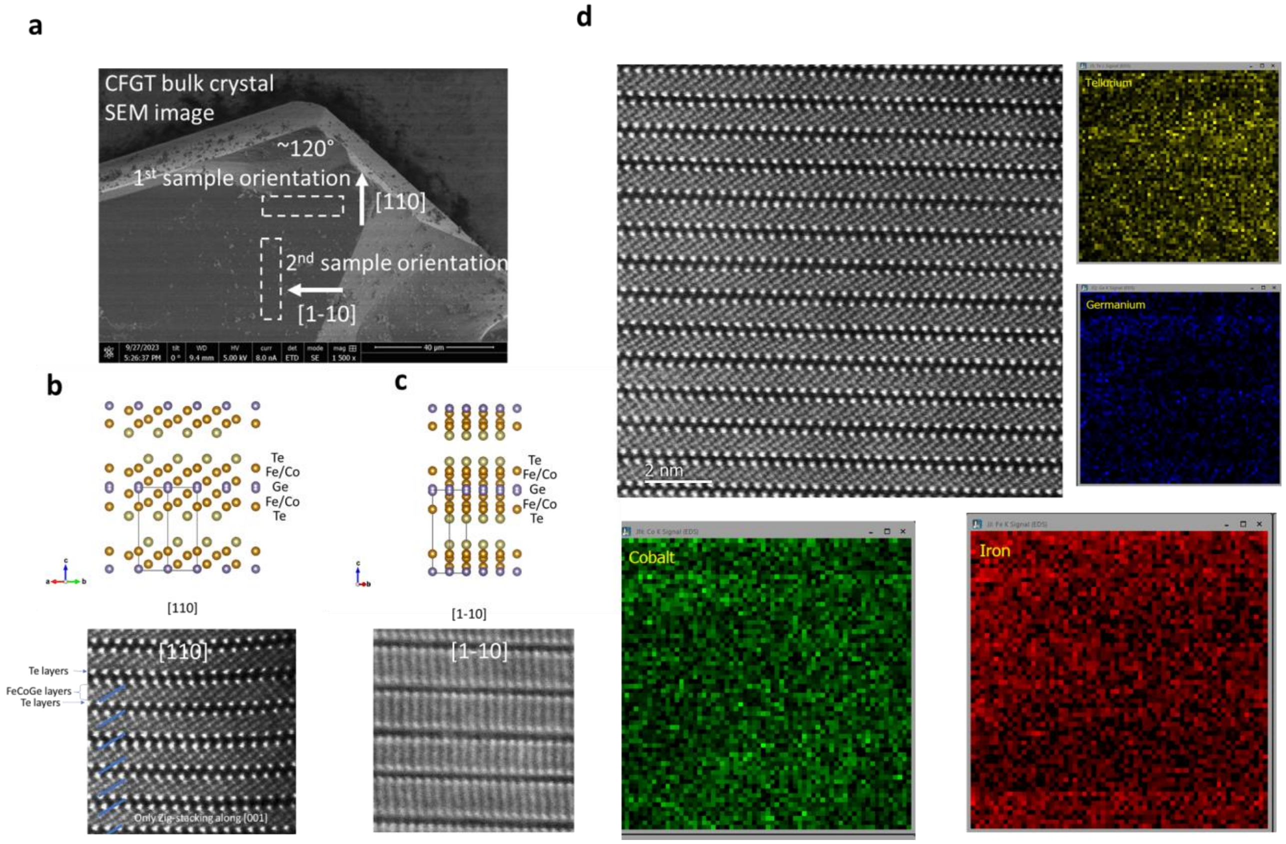

Supplementary Figure S1. STEM characterization of CFGT. a. *SEM image of the bulk CFGT crystal. Two specimens were prepared along [110] and [1-10].* ***b, c.*** *Atomic structure models projected along [110] and [1-10] and the corresponding HAADF STEM images.* ***d.*** *STEM images at a larger scale and the element analysis mapping of Co, Fe, Ge, and Te.*

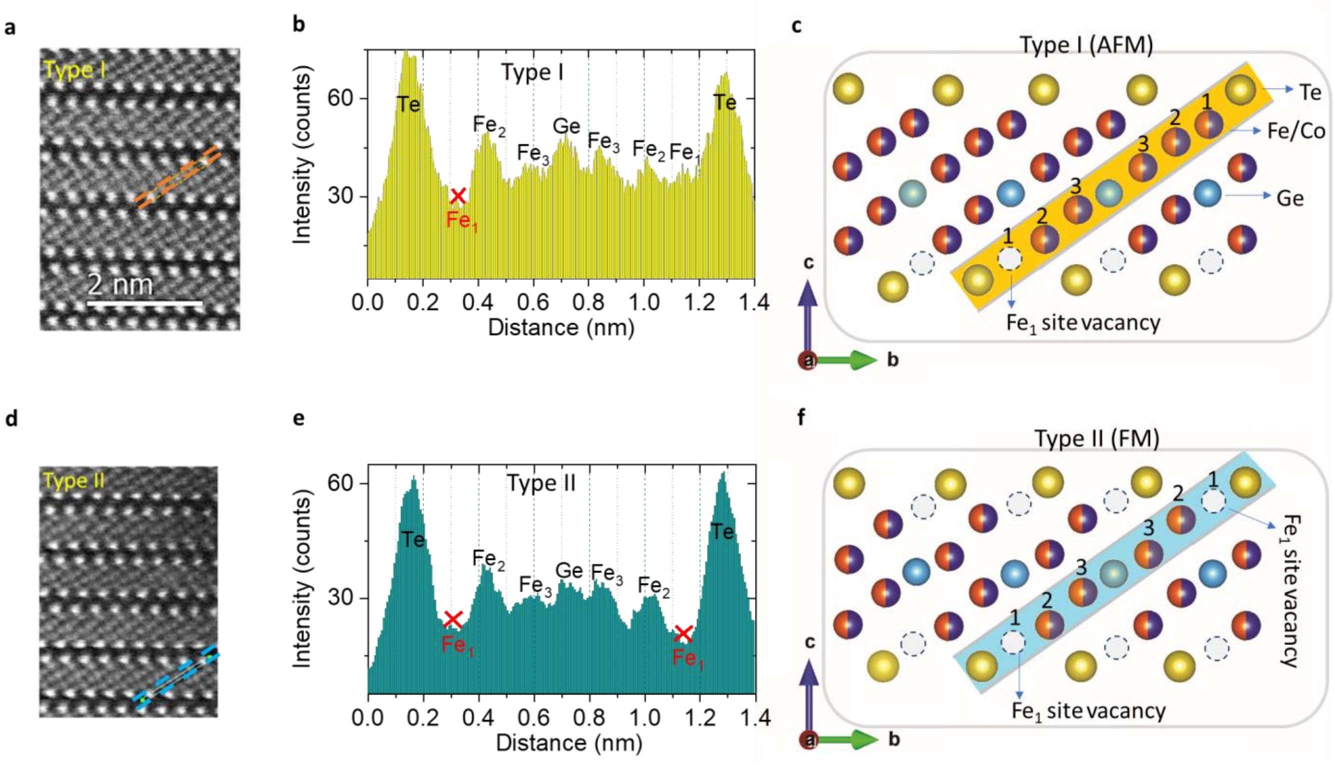

Supplementary Figure S2. Atomic structures of type I and II in CFGT. a,d. *Atomic resolution high-angle annular dark field (HAADF) scanning transmission electron microscopy (STEM) images of CFGT with Type I (II) crystal atomic stackings.* ***b,e.*** *Atomic profiles along the TeFe(Co)GeTe atom chain indicated with orange/blue dashed lines in the STEM images in* ***a, d)****. The peaks of the atomic profiles show the positions of individual atomic columns along the TeFe(Co)GeTe atom chain.* ***c,f.*** *Schematic illustrations of the crystal structures of Type I (II) atomic stacking, respectively. Fe_1-site vacancy (anti)symmetric distribution is represented by the dashed empty cycles.*

Table S1. Comparison with reference[2]

Property/Observation	Our CFGT	$(Co_xFe_{1-x})_5GeTe_2$
Atomic stacking	AA atomic stacking in both AFM and FM orders (due to Fe_1-site vacancy symmetry)	AA stacking (AFM) for x=0.45<br>AA' stacking (FM) for x=0.50
Sublattice configuration	AA: Parallel sublattices	AA: Parallel sublattices;<br>AA': Zig-zag distributed
Hall signals	Coexistence of AFM and FM Hall signals	AFM or FM Hall signals at two different Co-doping levels
Magnetic anisotropy	Canted FM order	Perpendicular Magnetic Anisotropy
Fe (Co) numbers	AFM: 5; FM 4	AFM: 6; FM 5
Magnetic orders and Phases	Two magnetic orders in one phase	One magnetic order in each phase

Supplementary Note 2. Discussion on the unique AA-stacked structures of CFGT

Compared to recent work[2], there are two different Fe1-site vacancies with (anti)symmetric atomic distribution CFGT. The Fe1-site vacancies are universal and energetically stable. However, both kinds of Fe1-

site vacancies share the same AA atomic stacking (space group $P\bar{3}m1$, No. 164). The (anti)symmetric atomic distribution makes it possible to form AFM and FM magnetic orders, respectively. The interaction between the AFM and FM orders induces a canted magnetization. This unique structure is neither a random mixing of two different phases of AA (AFM) and AA' (FM) nor the traditional AFM/FM stacking layers, which makes the material synthesis much easier compared to the traditional complicated interface engineering or some special phase mixing method.

Supplementary Note 3. DFT calculation results and Computational methods

DFT calculation results - DFT calculations were performed considering the structural characteristics obtained from STEM characterization (see Fig. S2). It can be inferred that the structures with five and six atoms between the two outermost Te sites correspond to Type I and Type II AA-stacked structures, respectively, as schematically represented in Fig. S2c and Fig. S2d. The sequence of Ge atoms and Co/Fe alloying sites are highlighted in slanted yellow boxes. Formation energies, presented in Table S1, show that the substitution of Co atoms in certain Fe sites is preferred. However, the distinctions in formation energies are not sufficiently pronounced to dismiss the potential for occupation in less favored sites. Accordingly, we anticipate a non-uniform distribution of Co atoms that modulates the magnetic properties[3]. To find the magnetic ground states for Type I and Type II, we generated one hundred random alloys of each structural phase and calculated the energy differences between AFM and FM configurations. Boxplots in the main text Fig. 2a illustrate these structural dependencies of magnetic ground states for Type I and Type II, where each dot represents a distinct Co distribution. Although AFM and FM states coexist in both Type I and Type II, their distribution is non-uniform. In Type II, the FM intra-layer coupling is in the majority, whereas, in Type I, AFM inter-layer coupling is predominant. These findings imply that the coexistence of Type I and Type II atomic structures can result in the coexistence of ferromagnetic and antiferromagnetic orders in our samples. The magnetic moments on Fe and Co atoms also depend on their respective sites. On average, Co atoms possess a magnetic moment of 0.93 ±0.21 μ_B, while Fe atoms exhibit a magnetic moment of 2.04 ± 0.70 μ_B. This implies that the spin configuration of Fe atoms may vary depending on their surroundings[4].

Supplementary Table. S2. Formation energies of Co doping in various Fe sublattices in Type I and Type II CFGT. Here, negative numbers represent spontaneous formation energies.

Type I	Fe1	Fe2	Fe3	Fe4	Fe5
Formation energy (eV/Co)	-0.34	0.30	-0.16	-0.25	0.18

Type II	Fe1	Fe2
Formation energy (eV/Co)	0.15	-0.14

DFT Computational methods - We conducted structural optimization and computed magnetic ground states using the Vienna Ab initio Simulation Package (VASP)[5] based on plane wave basis set and projector augmented wave pseudopotentials. The exchange-correlation potential was described using the generalized gradient approximation (GGA) with the Perdew, Burke, and Ernzerhof (PBE) functional[6,7]. For Brillouin zone integration, a 15 × 15 × 3 k-point grid was employed for bulk CFGT in AA stacking. We incorporated van der Waals corrections using the DFT-D3 method developed by Grimme[8]. Equilibrium lattice constants and atomic positions were obtained via energy minimization, employing the conjugate gradient method until the force components on each atom fell below 0.01 eV/Å. Formation energies were computed relative to bulk BCC Fe and HCP Co. The structures optimized with VASP served as the basis for calculating interatomic magnetic exchange interactions (J_{ij}). The magnetic properties were computed using the QuantumATK-Synopsys package version U-2022, employing an LCAO basis set and the "PseudoDojo" pseudopotential[9,10]. Self-consistent calculations utilized a density mesh cutoff of 140 Hartree and a 15 × 15 × 3 k-point grid. For the determination of magnetic anisotropy energy and isotropic Heisenberg exchange interaction parameters, a finer k-point grid of 25 × 25 × 5 was employed. The magnetocrystalline anisotropy energy was evaluated using the force theorem, utilizing the expression

$$MAE = E_{\parallel} - E_{\perp} \qquad \text{(Eq. S1)}$$

Where $E_{\parallel}$ and $E_{\perp}$ denote total energies for perpendicular and in-plane magnetism, respectively in the presence of spin-orbit coupling, accordingly, a positive MAE value shows a perpendicular out-of-plane easy axis.

The extracted J_{ij} interaction parameters were incorporated into a Heisenberg Hamiltonian to determine the magnetic order through classical Monte Carlo (MC) simulations using the UppASD code[11]. To ensure properly averaged properties, calculations were performed in a supercell with dimensions of 20 × 20 × 5 under periodic boundary conditions. The Monte Carlo simulations utilized the following spin-Hamiltonian:

$$H = -\sum_{i \neq j} J_{ij} e_i . e_j - \sum_i K_i (e_i^z)^2 \qquad \text{(Eq. S2)}$$

Here, J_{ij} represents the isotropic symmetric exchange coupling between magnetic moments at sites *i* and *j*, e_i denotes the unit vector along the direction of the magnetic moment at site i, and $J_{ij}>0$ (<0) indicates ferromagnetic(antiferromagnetic) coupling. K_i denotes the strength of single-ion uniaxial magneto-crystalline anisotropy energy for site *i*, obtained by dividing the total MAE per cell by the number of magnetic atoms in the unit cell.

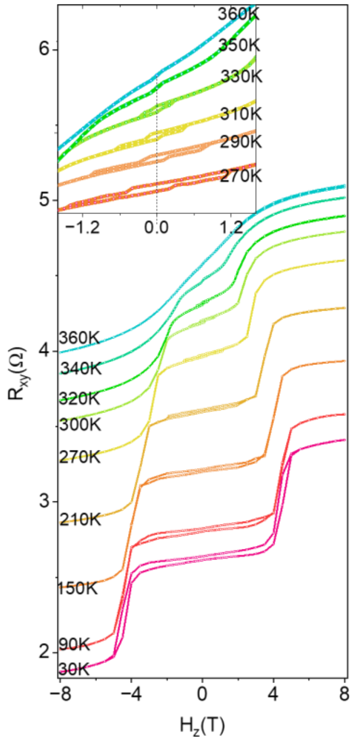

Supplementary Figure S3. *Temperature dependence of the AHE in CFGT nanolayers in the range of 30-360 K. The different magnetic phases were realized by applying an external field of H_z. FM0 and AFM phase are distinguished by the magnetization coercive field H_c of the magnetic hysteresis loop and AFM Néel vectors realignment process (transition FM, TFM). With increased H_z, all the magnetic orders finally align along the z-axis to form a single magnetic order FM1. To be noted, AFM and FM0 coexist at a small field range up to around 360 K.*

Supplementary Note 4. Extraction of the magnetization canted angle ΔΦ in CFGT

The formula $H_{c0} = H_c\cos(\Phi-\Delta\Phi)$ is a geometric mathematical relation between the nominal coercivity Hc(Φ) and the field sweep angles Φ. H_{c0} is the intrinsic coercivity when the field is aligned with the magnetic easy axis. To make sure the formula is applicable, the single magnetic domain M and remaining along its easy axis requirements should be satisfied based on the following arguments: (1) In the small field range, the AHE signal magnitude vs. field angle Φ curve (main text Fig. 2d) shows no clear change at most angles. This suggests the magnetization M is barely pulled away from the easy axis, but switching between +M and -M given the field range here at all the field sweep angles Φ. Furthermore, the field range applied (+/- 0.6T) is ignorable compared

to the AFM order and the perpendicular anisotropy field H_k (magnetic hard axis) for the FM order (see Fig.S3a). (2) This formula, as an approximation, applies to small titled angles of the magnetic easy axis with a smaller Hall device (~1x1 μm^2), which usually hosts a single domain. (3) The exchange anisotropy is unidirectional[12], so it produces one easy direction of magnetization. These arguments confirm that the canted angle extraction method applies to the geometric mathematical relation.

We then formulate the relation between the field angle Φ dependent exchange bias field H_{EB} (see inset in Fig. S4b). $H_{EB}=(H_{c+}+H_{c-})/2=0.5*(H_{c0+}+H_{c0-})/\cos(\Phi-\Delta\Phi)=H_{EB0}/\cos(\Phi-\Delta\Phi)$, where $H_{EB0}=(H_{c0+}+H_{c0-})/2$ is the intrinsic exchange bias field along the easy axis of magnetization defined by CFGT. By fitting the experimental results for H_{EB}, we obtain the canting angle of the magnetization with respect to the z-axis as $\Delta\Phi=10^{o}\pm4^{o}$ (see main Fig.2d). The reproduced result in another CFGT flake of $\Delta\Phi=11^{o}\pm3^{o}$, as shown in Fig. S4.

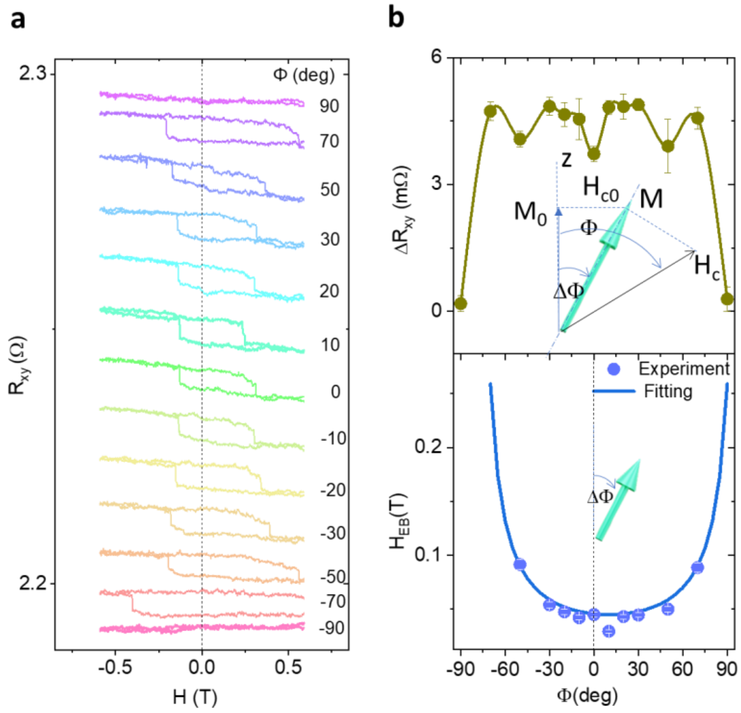

Supplementary Figure S4. Canted perpendicular magnetic anisotropy of CFGT. a, b. *Reproducibility of the angle dependence of AHE in CFGT and the extracted AHE signal magnitude ΔR_{xy} and exchange bias field H_{EB} with a fitting to the experimental results. The insets show the relation between the canted magnetization M and external field H_c at an angle Φ. The reproduced result in another CFGT flake of $\Delta\Phi=11^{o}\pm3^{o}$.*

Supplementary Note 5. The origin of the canted magnetization

Theoretical calculations indicate that the magnetocrystalline anisotropy energy (MAE) and the interplay between magnetic exchange interactions may give rise to the canted angle observed in CFGT[13,14]. According to our DFT calculations, both AA-stacked undoped Type I and Type II exhibit in-plane MAE of -0.37 and -2.58

meV/f.u., respectively. Further analysis of atom-resolved MAE (Fig. S5 and Fig. S6) reveals that although the in-plane tendency generally originates from Fe atoms, MAE on different sublattices is not equivalent. A transition from in-plane to perpendicular MAE may occur, depending on which sublattices are occupied by Co atoms. Additionally, Co-doping in one site also influences the MAE on neighboring sites. We found that the substitution of Co atoms, in the most energetically favored sites, results in perpendicular MAEs of 1.70 and 3.10 meV/f.u. for Type I and Type II, respectively (see atom-resolved MAEs in Fig. S5 and Fig. S6). These findings suggest that the direction and magnitude of MAE heavily depend on the local Co concentration and their distribution. This indicates that these potential differences in local MAE contribute to the overall canted angle observed in our samples.

Another contributing factor is the competition between exchange interactions between J_{ij} and MAE. Monte Carlo simulations were conducted to investigate this competition and gain insight into the magnetic texture CFGT. As schematically depicted in Fig. S6d, exchange interactions in Type II, introduced by the exchange interaction between FM and AFM magnetic orders, on average tilt the magnetic moments by approximately 8° from the z-axis. The canted axis is dependent on exchange interactions, and magnetic anisotropy energy. As these parameters are dependent on the structure and composition, different calculations give different canted angles along the bc plane ([1-10] plane). However, we believe that on average they should align with the experimental results, which is as indicated in Fig. S6d and Fig. S2d. It is important to note that these two specific Co distributions may not encompass all possible alloying configurations. Nevertheless, they effectively show that the interplay between J_{ij} and MAE can play a critical role in establishing canted magnetism

Another possible factor for the tilted magnetization anisotropy can be due to the Dzyaloshinskii-Moriya Interaction (DMI)[15,16] in the sample. Such DMI interaction should show an apparent topological Hall effect (THE)[17] in the anomalous Hall effect signals. However, we do not see any signature of THE in the anomalous Hall effect signal in CFGT/Pt (Fig. 3b in the main text) and single CFGT (Fig. 2e in the main text) Hall bar devices. These observations suggest that the role of DMI for the tilted magnetism in CFGT may not be a key factor in our devices.

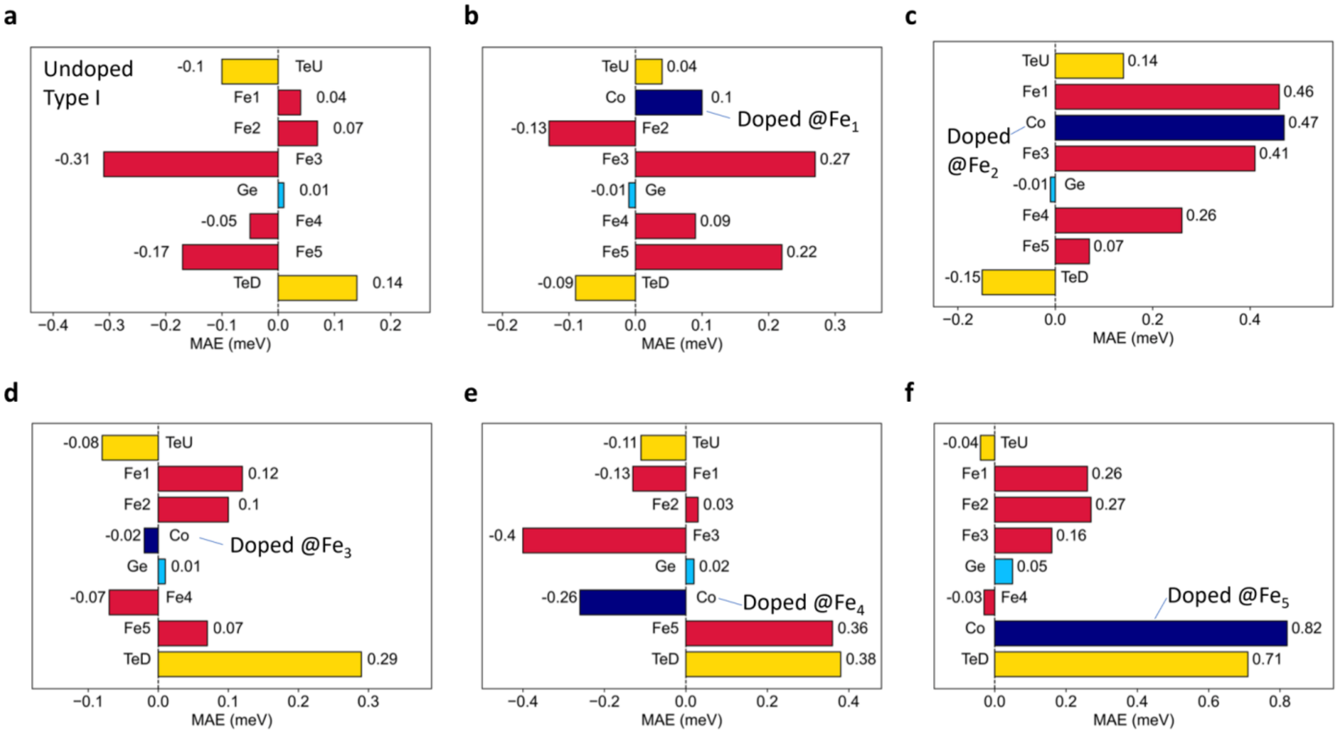

Supplementary Figure S5. a-f. *Atom-resolved MAEs are depicted for undoped Type I (a), Type I doped in the Fe_1 sublattice (b), Type I doped in the Fe_2 sublattice (c), Type I doped in the Fe_3 sublattice (d), Type I doped in the Fe_4 sublattice (e), and Type I doped in the Fe_5 sublattice (f).*

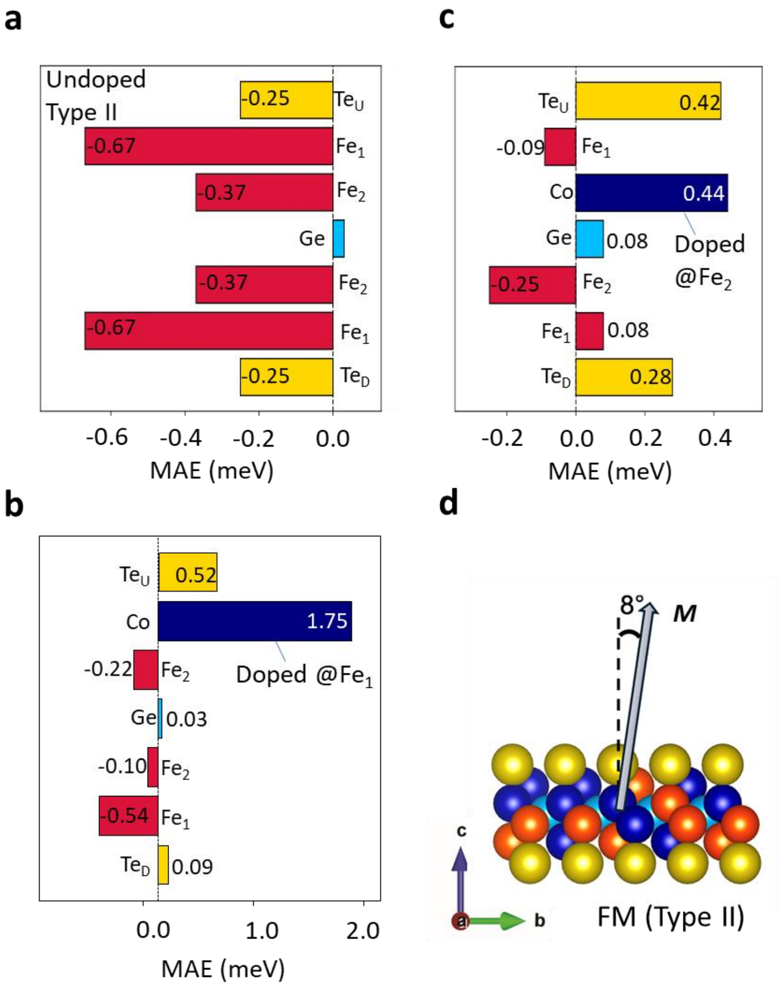

Supplementary Figure S6. a-c. *An atom-resolved MAE is depicted for undoped Type II, Co-doped in the Fe_1 sublattice of Type II, and Co-doped in the Fe_2 sublattice of Type II.* ***f.*** *The averaged canted angle for AA-stacked Type II extracted from Monte Carlo simulation.*

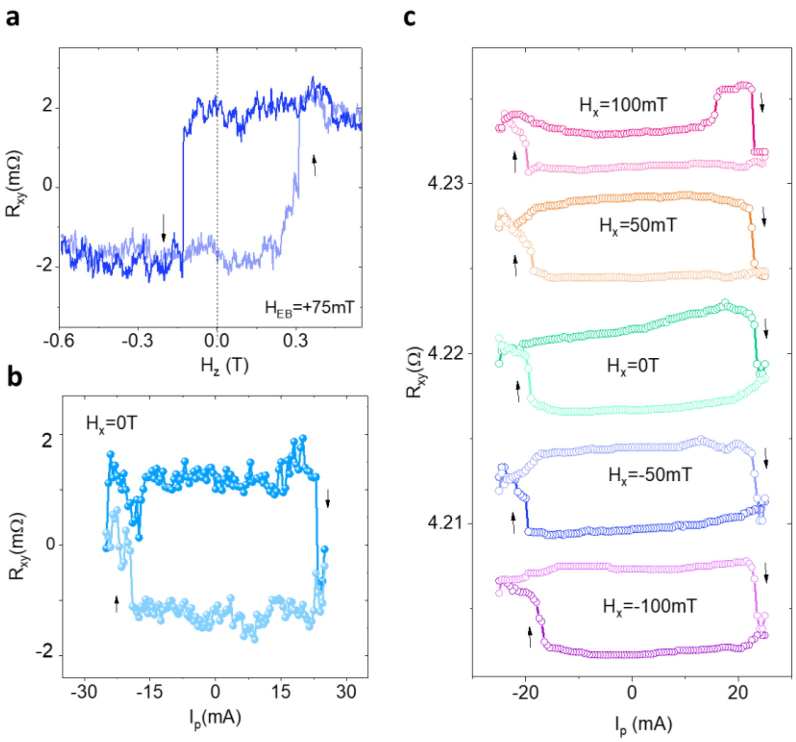

Supplementary Figure S7. Field-free spin-orbit torque magnetization switching in CFGT/Pt heterostructure at room temperature in Dev 3. a. *AHE signals with smaller exchange field H_{EB}=+75mT.* ***b.*** *Pulsed write current I_p induced a transverse Hall signal R_{xy} change due to SOT-induced magnetic switching without an external magnetic field.* ***c.*** *The measured SOT magnetization switching signals with different in-plane fields H_x.*

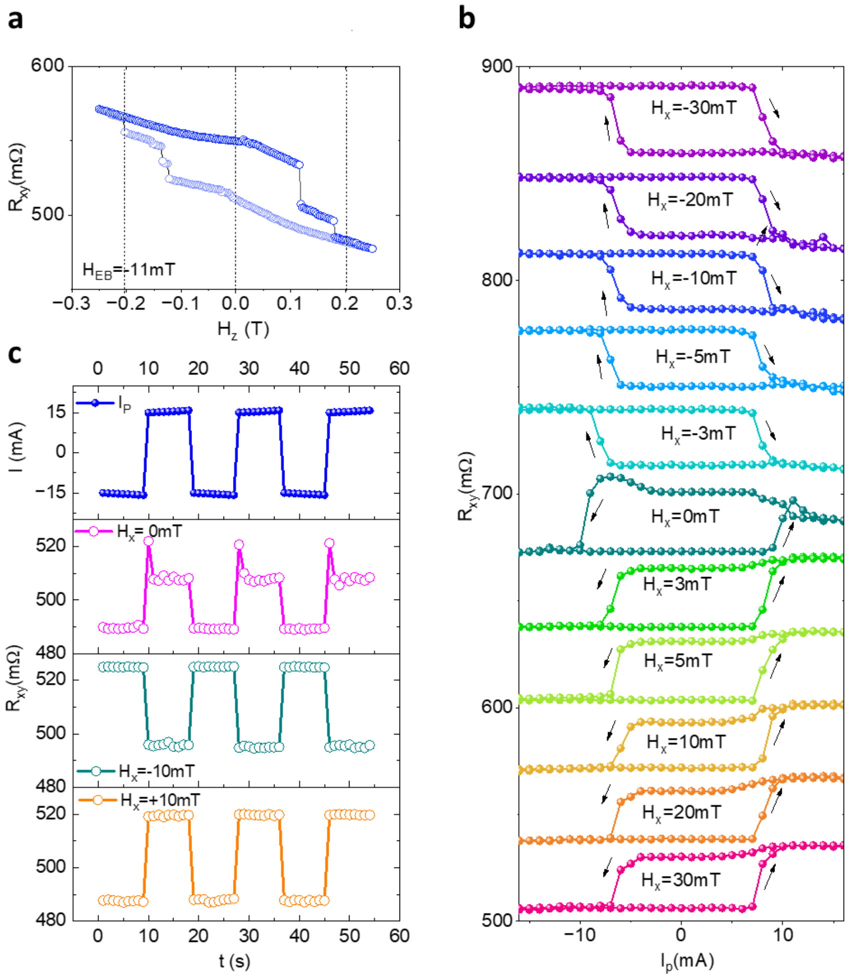

Supplementary Figure S8. Field-free spin-orbit torque magnetization switching in CFGT/Pt heterostructure at room temperature in Dev 4. a. *AHE signals with smaller exchange field H_{EB}=-11mT.* ***b.*** *The measured SOT magnetization switching signals with different in-plane fields H_x.* ***c.*** *Time dependence of the pulse current I_p and the corresponding measured AHE signal R_{xy} at in-plane fields H_x=0 and ±10 mT.*

Supplementary Note 6. Origin of the field-free SOT-induced magnetization switching

(1) **Effect of Oersted field on SOT switching -**To compete with the coercive field of CFGT, the Oersted field magnitude should be comparable. In our case, the Oersted field is around 0.9 mT at 20 mA, which is much smaller than the coercive field (~0.1 T) of CFGT. Oersted field may help to nucleate longitudinal magnetic domains of opposite directions on each side of the current path, however, it is not possible to switch the magnetization. Furthermore, the pulse current induced Oersted field direction is defined by the right-hand screw rule, which circles about the current direction. This cannot work as an effective field in x-direction. Therefore, the current-induced Oersted field here cannot be the origin of the observed field-free SOT-induced magnetization switching[18].

(2) Canted magnetism as the origin of the field-free SOT-induced magnetization switching - The current-induced spin polarization S_y, originating from the spin Hall effect, exerts two typical effective torques (damping-like torque τ_{DL} and field-like torque τ_{FL}) on the magnetization M[19]. For ultrahigh-density storage, the perpendicular magnetic anisotropy (PMA) is preferred (Fig. S9b). However, due to the intact geometry symmetry, the effective torques can only rotate M toward the in-plane direction (Fig. S9b, step 2), which leaves the M in an undetermined state when the effect torques are removed (Fig. S9b, step 3). In other words, M has an equal chance to rotate to +M or -M. To obtain a deterministic switching of M, an additional external field H_x is needed to assist the switching (Fig. S9c)[20].

From a practical perspective, people have experimented with various methods to eliminate the external field to achieve field-free SOT-induced magnetization switching. As shown in the main Fig. 4e, this involves canted magnetism, z-spin polarization, lateral asymmetry, and so on.

Here, CFGT presents a canted magnetism due to the MAE and strong exchange bias effect between AFM and FM orders. In such a scenario, the canted M with a titled hard axis (Fig. S9a) breaks the geometry symmetry for the switching. The damping-like torque τ_{DL} solely can rotate M over the hard magnetic axis toward -M without an external field, to realize a deterministic switching of M. Therefore, the field-free SOT switching is attributed to the canted magnetization M in CFGT.

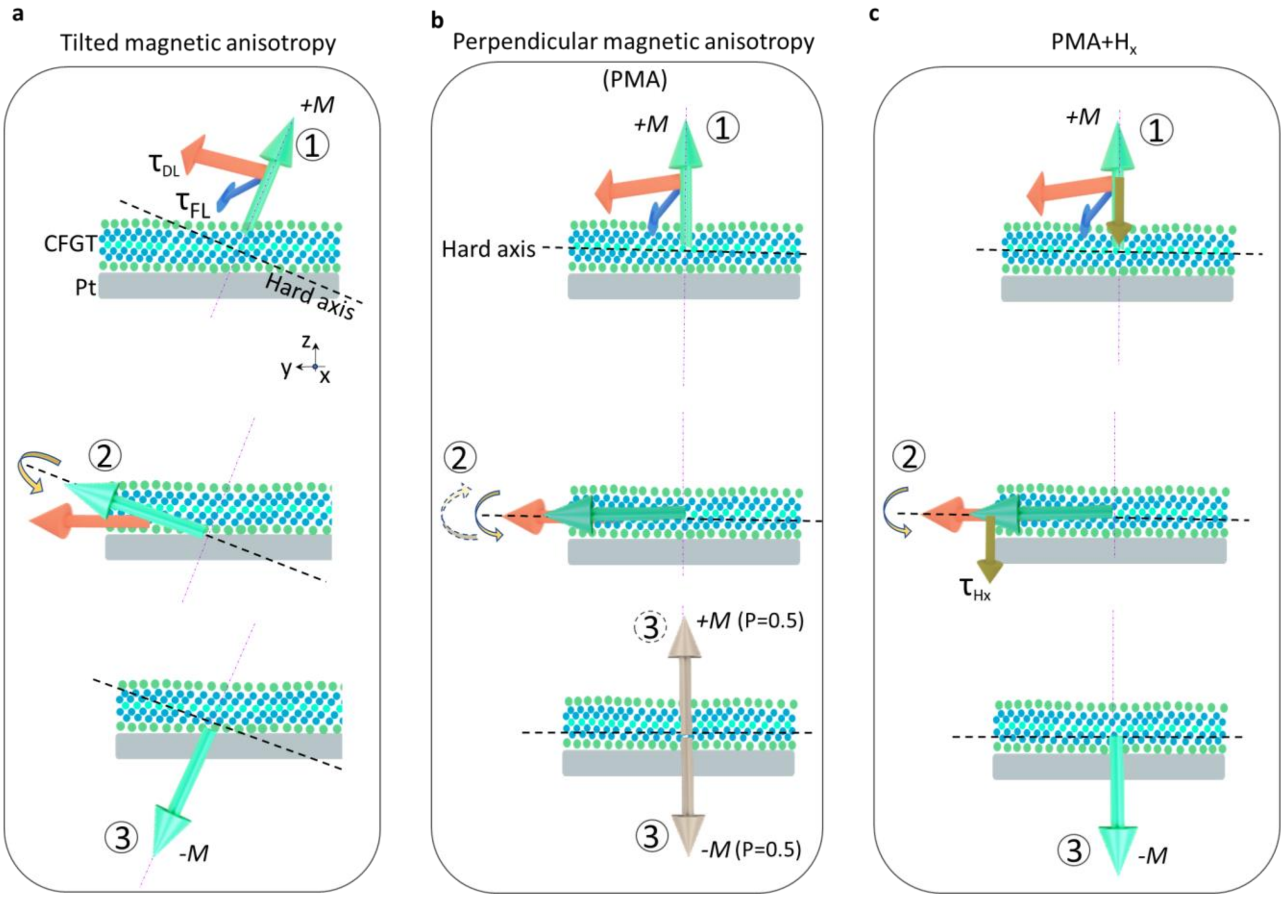

Supplementary Figure S9. Origin of the field-induced magnetization switching. a. *Mechanism of the field-free SOT-induced the canted magnetization (M) switching of CFGT in three key steps. The dumpling-like torque τ_{DL} solely rotates the M to another easy axis.* ***b, c.*** *Mechanism of the traditional SOT-induced PMA magnetization switching without/with external field H_x. The H_x-induced additional torque τ_{Hx} leads to the deterministic switching.*

Supplementary Note 7. Harmonic Hall measurements for SOT quantification with the canted magnetization and exchange bias effect in CFGT/Pt heterostructure devices

1. Influence of the canted magnetic easy axis and exchange bias effect on the harmonic Hall measurements – The 2nd harmonics theories on SOTs have been well established by the pioneer works[21,22]. This method is valid for systems with perpendicular magnetic anisotropy provided that the magnetization is pulled into the plane with an external field larger than the effective perpendicular anisotropy field H_k[22]. This method has been adopted by many works in literature with PMA systems[22–24]. According to the 1st Harmonic signal as a function of the H_x (main Fig. 4a), one can tell that magnetization can be fully pulled into the xy-plane at around 2T; while our measurements were performed at H=6~10T. Therefore, the canted magnetic

easy axis and exchange bias are still applied for both the angle (Eq.1 in the main text) and field (Eq. S3) dependence harmonics measurements.

The 2nd harmonic Hall voltage signal as a function of the large longitudinal in-plane field H_x includes the contributions from damping-like effective field H_{DL}, field-like effective field H_{FL}, and thermal-related effect as below[25],

$$V_{xy}^{2\omega} = V_{AHE}^{2\omega} + V_{PHE}^{2\omega} + V_{thermal}^{2\omega}\frac{H_x}{|H_x|} + c$$

$$= \frac{V_{AHE}}{2}\frac{H_{DL}}{|H_x| - H_k} + V_{PHE}\frac{H_{FL}}{|H_x|} + V_{thermal}\frac{H_x}{|H_x|} + c; \quad \text{(Eq. S3)}$$

We can extract the effective fields and thermal-related contribution $V_{thermal}$ by fitting the curve in Fig. S10d and Fig. S10c.

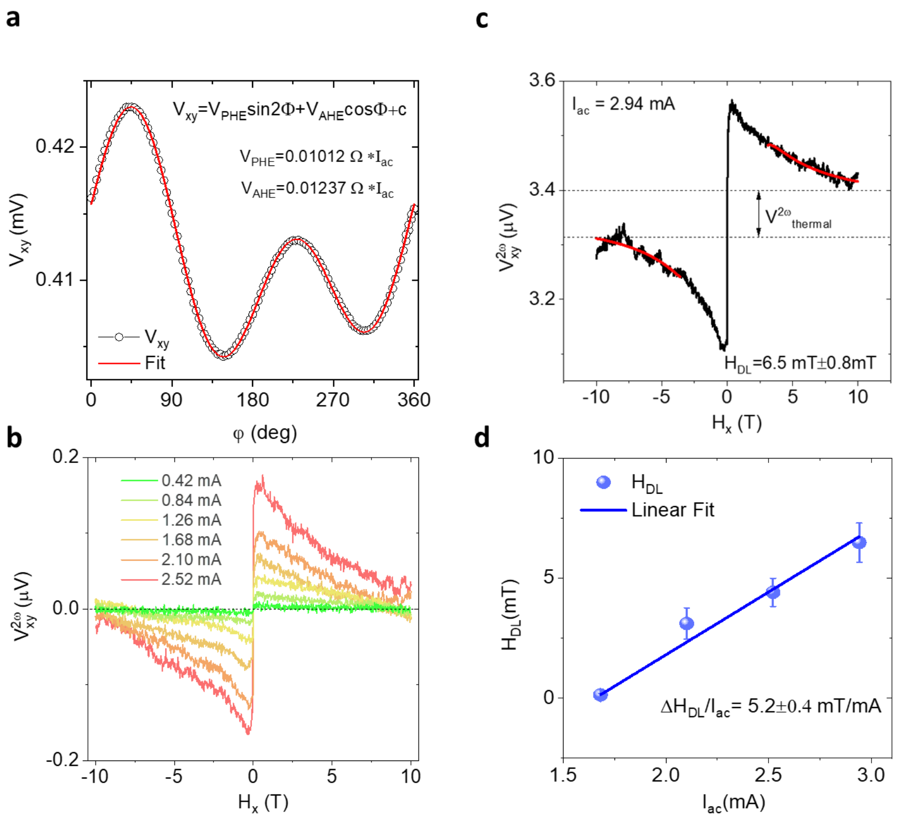

Supplementary Fig. S10. Extraction of the effective fields and thermal contributions in the field dependent harmonic measurements. a. *In-plane angle dependence of the V_{xy} (DC method) at a large fixed in-plane field H_{ip}=8 T and the fitting result with a formula including contribution from AHE and PHE.* ***b, c.*** *The 2nd harmonic Hall voltage signal as a function of the large longitudinal in-plane field H_x and fitting results with Eq. S6 at different bias currents indicated.* ***d.*** *Bias dependence of the extracted effective field H_{DL} with a linear fitting result $\Delta H_{DL}/I_{ac}$=5.2 mT/mA in Dev 5.*

As the damping-like torque is attributed to the longitudinal effective spin-orbit field H_{DL}, the spin-orbit torque efficiency can be calculated using the expression[26,27],

$$\xi_{SOT}=T_{ini}\theta_{SH}=\frac{2e}{\hbar}\mu_0 M_s t_{CFGT}^{eff}\Delta H_{DL}/J_{ac}\,; \qquad \text{(Eq. S4)}$$

where e is the electron charge, $\hbar$ is the reduced Plank constant, $\mu_0 M_s$ and t_{CFGT}^{eff} are the saturation magnetization (net magnetization at low field range) and the effective thickness of CFGT, respectively. Here ξ_{DL} is determined by the spin Hall angle (θ_{SH}) of Pt and the interface transparency (T_{ini}). By assuming a fully transparent interface, i.e. T_{ini}=1, and spin Hall angle θ_{SH}=0.12 for Pt[27–29], we obtain the effective saturation magnetization $\mu_0 M_s$ ≈3.3 emu/cm^3 (see more detailed results of the different devices in Supplementary Table 3).

To be noted, the extracted $\mu_0 M_s$ and critical current density J_{sw} from different devices (see Supplementary Table S3), have some sample dependence due to the CFGT thickness. The effective field H_{DL}/J_{ac} is larger in thinner CFGT nanolayers (Correspondingly, $\mu_0 M_s$ shows the opposite trend due to the inversely proportional relation in the expression of ξ_{SOT}). It agrees with the smaller critical current density needed to switch thinner CFGT nanolayers as well. This is true if we consider a spin diffusion length (λ_s) of ~nm to tens of nanometers in the FM materials[30,31]. As a spin current transfer from spin-orbit materials to adjacent ferromagnets is typically limited by λ_s. The thicker of the FM material, the less effective the SOT field should be extracted. Therefore, the extracted SOT parameters are the lower (upper) limit for the effective fields (critical switching current density) if thinner CFGT devices are considered.

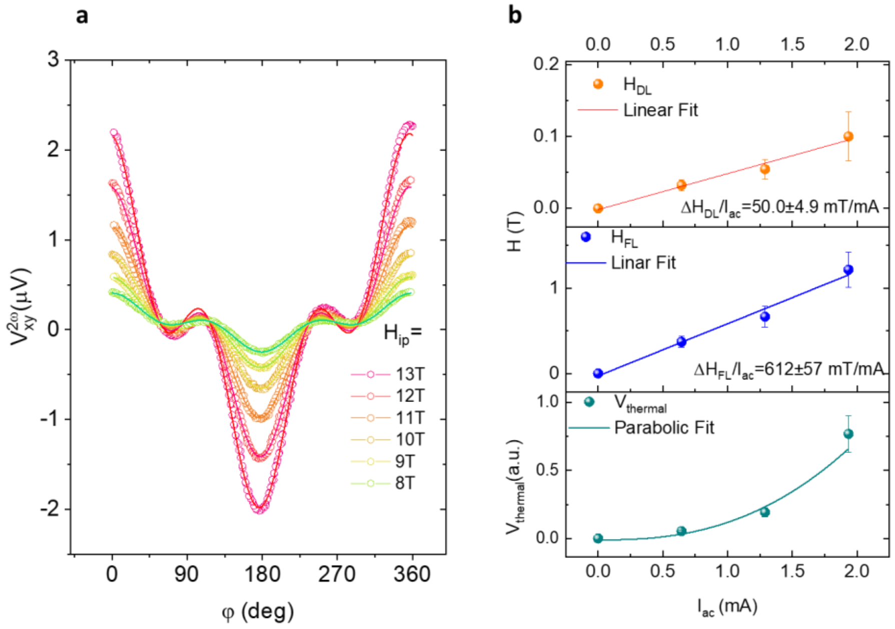

Supplementary Fig. S11. In-plane rotation harmonic measurement to extract the effective fields and thermal contributions in Dev 6. a. *In-plane angle dependence of the 2nd Harmonic signal at I_{ac}=1.3 mA with in-plane field H_{ip}. The sign opposite to the results of Dev 2 is due to $Ø_{lockin}$ = -90 deg here. The solid curves are the fitting results with Eq. 1 of the main text.* ***b.*** *Extracted current dependence of the field-like effective field H_{FL}, damping-like effective field $H_{DL,}$ and thermal contribution.*

Supplementary Note 8. State of the art of van der Waals magnet-based SOT devices

Our work reports the SOT phenomenon originating from the unique atomic crystal structure of CFGT. These features have not been observed before despite numerous research efforts on vdW magnet materials (Fig. S12). This implies that vdW magnets may exhibit a completely different phenomenology in a single material, going beyond the thin limits of conventional magnets with clean interfaces. Lastly, our work provides a unique way to tune the magnetism for vdW magnets that cannot be achieved by conventional magnets.

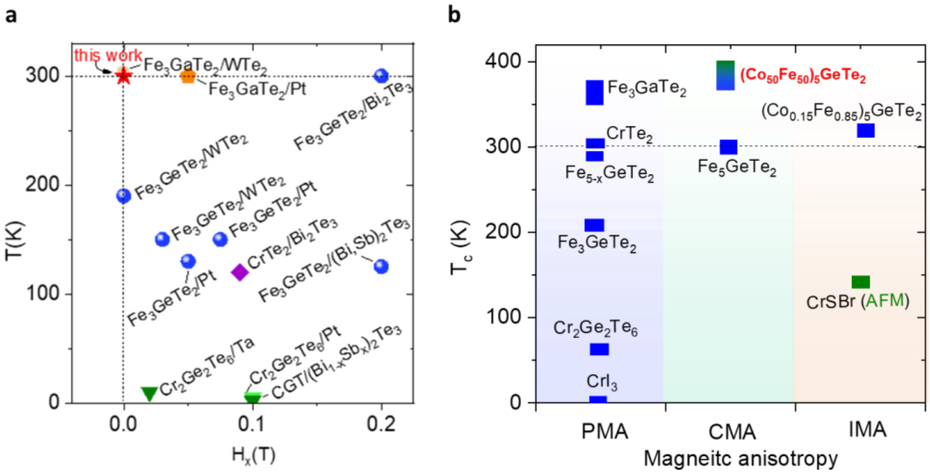

Supplementary Fig. S12. a. *Benchmark plot showing the state-of-the-art of the vdW magnets-based SOT devices, considering working temperature vs. in-plane assistant magnetic field H_x. Benchmarking our work with state-of-the-art results using vdW magnet-based SOT devices of Fe_3GeTe_2/WTe_2[32], Fe_3GeTe_2/WTe_2[33], Fe_3GeTe_2/Pt[28], Fe_3GeTe_2/Pt[29], $Cr_2Ge_2Te_6$/Ta[34], $Cr_2Ge_2Te_6$/Pt[35], $Cr_2Ge_2Te_6/(Bi_{1-x}Sb_x)_2Te_3$[36], FeGaTe/Pt[37], FeGaTe/WTe_2[38] . Our work on the CFGT/Pt device shows room-temperature SOT device operation without any assisted magnetic fields.* ***b.*** *State of the art of the 2D magnet Cuire temperature as a function of magnetic anisotropy (PMA: perpendicular magnetic anisotropy; CMA: canted magnetic anisotropy; IMA: in-plane magnetic anisotropy).*

Supplementary Table S3. Key SOT parameters of the measured CFGT/Pt devices

The key parameters of CFGT/Pt SOT Hall devices: material thickness t_{CFGT}, t_{Pt}=10 nm, and resistivities ρ_{CFGT}=1*10^{-6} Ohm*m and ρ_{Pt}=4*10^{-7} Ohm*m. The CFGT/Pt Hall channel width W and length L; the calculated shunting current in Pt out of the total device ratio=I_{Pt}/I_{tot} based on the parallel resistors model. Damping (Field)-like $\Delta H_{DL(FL)}/J_{ac}$ effective fields; effective saturation magnetization $\mu_0 M_s$. Critical switching current density J_{sw}; Different SOT characterization methods are noted in the method section, with 2nd harmonics and pulse switching method, respectively.

Devices	t_{CFGT} (nm)	W (μm)	ratio= I_{pt}/I_{tot}	$\Delta H_{DL}/J_{ac}$ (mT per MA/cm^2)	$\Delta H_{FL}/J_{ac}$ (mT per MA/cm^2)	$\mu_0 M_s$ (emu/cm^3)	J_{sw} (MA/cm^2)	method
Dev 1	40	2.7	0.38				9.8	Pulse Switching
Dev 2	45	2.6	0.36	4.2	19.7	2.1		2nd harmonics
Dev 3	40	3.8	0.38				-	Pulse Switching
Dev 4	35	5.1	0.42				8.2	Pulse Switching
Dev 5	50	1.5	0.33	2.4	-	3.3		2nd harmonics
Dev 6	20	2.7	0.48	19.5	141.4	1.0		2nd harmonics

Supplementary Note 9. Ruling out the possibility of the exchange bias effect caused by oxidation on the surface layer

Our CFGT with 50% Co doping, presents the dominant AFM orders and a smaller finite FM state arising from the presence of symmetric Fe vacancies. Through STEM and DFT, we reveal that the dominant AFM component exhibits an anti-symmetric Fe_1 site vacancy (type I) atomic structure, while the FM orders are introduced by the additional symmetric Fe_1 site vacancies (type II).

The oxidation of FM surface can introduce an AFM order and exchange bias effect that has been reported before, like in Fe_3GeTe_2[39] and Cr_5Te_6[40]. This effect is observed with intentional oxidation of the surface layers, where the base material is an FM, and the oxide is an antiferromagnet. However, in our case, the base material CFGT is AFM, and a small FM order comes from Fe/Co vacancies.

Furthermore, in our case, (i) we have around 2 nm Al_2O_3 as a caplayer on CFGT, which should prevent the oxidation of the CFGT. However, any such small oxidation layer cannot explain a giant AFM magnetic order portion of ~90% compared to small FM order of ~90% in CFGT (see Fig. S3). (ii) Typically, these surface oxidation-induced AFM/FM exchange effects should not show AFM behavior in the AHE signals, which is due to the electrical insulating character of the oxide layer; while our CFGT predominantly shows the AFM behavior, i.e. a benchmarking “three-stage” AHE signal, which is due to the spin flop of the Néel vector with external magnetic field.

So, both the AFM signal and exchange effects in our CFGT are due to the coexistence of FM and AFM states in CFGT, not the oxidation of the surface layers. Our CFGT is mainly AFM with FM status coming from the presence of the symmetric Fe vacancies. This agrees with previous works with 44%-45% Co doping CFGT typically showing AFM magnetic orders with AA phase[2,41]. Our CFGT with 50% Co doping, presents the dominant AFM magnetic orders and finite FM orders. Through STEM and DFT calculations, we reveal that AFM

and FM orders in our CFGT share the AA structure (i.e. the parent material structure). The dominant AFM component exhibits an anti-symmetric Fe_1 vacancy (type I) atomic structure, while the FM orders are introduced by the additional symmetric Fe_1 vacancies (type II).

Supplementary References

1. May, A. F., Du, M.-H., Cooper, V. R. & McGuire, M. A. Tuning magnetic order in the van der Waals metal Fe5GeTe2 by cobalt substitution. *Phys. Rev. Mater.* **4**, 074008 (2020).
2. Zhang, H. *et al.* A room temperature polar magnetic metal. *Phys. Rev. Mater.* **6**, 044403 (2022).
3. Ghosh, S., Ershadrad, S. & Sanyal, B. Structural distortion and dynamical electron correlation driven enhanced ferromagnetism in Ni-doped two-dimensional Fe5GeTe2 beyond room temperature. *2D Mater.* **11**, 035002 (2024).
4. Ghosh, S., Ershadrad, S., Borisov, V. & Sanyal, B. Unraveling effects of electron correlation in two-dimensional FenGeTe2 (n = 3, 4, 5) by dynamical mean field theory. *npj Comput. Mater.* **9**, 86 (2023).
5. Kresse, G. & Joubert, D. From ultrasoft pseudopotentials to the projector augmented-wave method. *Phys. Rev. B* **59**, 1758–1775 (1999).
6. Kresse, G. & Furthmüller, J. Efficient iterative schemes for ab initio total-energy calculations using a plane-wave basis set. *Phys. Rev. B* **54**, 11169–11186 (1996).
7. Perdew, J. P., Burke, K. & Ernzerhof, M. Generalized Gradient Approximation Made Simple. *Phys. Rev. Lett.* **77**, 3865–3868 (1996).
8. Grimme, S., Antony, J., Ehrlich, S. & Krieg, H. A consistent and accurate ab initio parametrization of density functional dispersion correction (DFT-D) for the 94 elements H-Pu. *J. Chem. Phys.* **132**, 154104 (2010).
9. Smidstrup, S. *et al.* QuantumATK: an integrated platform of electronic and atomic-scale modelling tools. *J. Phys. Condens. Matter* **32**, 015901 (2019).
10. van Setten, M. J. *et al.* The PseudoDojo: Training and grading a 85 element optimized norm-conserving pseudopotential table. *Comput. Phys. Commun.* **226**, 39–54 (2018).
11. Eriksson, O., Bergman, A., Bergqvist, L. & Hellsvik, J. *Atomistic spin dynamics: foundations and applications*. (2017).
12. Meiklejohn, W. H. & Bean, C. P. New Magnetic Anisotropy. *Phys. Rev.* **105**, 904–913 (1957).
13. Zhao, B. *et al.* A Room-Temperature Spin-Valve with van der Waals Ferromagnet Fe5GeTe2/Graphene Heterostructure. *Adv. Mater.* **35**, 2209113 (2023).
14. Ershadrad, S., Ghosh, S., Wang, D., Kvashnin, Y. & Sanyal, B. Unusual Magnetic Features in Two-Dimensional Fe5GeTe2 Induced by Structural Reconstructions. *J. Phys. Chem. Lett.* **13**, 4877–4883 (2022).
15. Thiaville, A., Rohart, S., Jué, É., Cros, V. & Fert, A. Dynamics of Dzyaloshinskii domain walls in ultrathin magnetic films. *EPL (Europhysics Lett.* **100**, 57002 (2012).
16. Fernández-Pacheco, A. *et al.* Symmetry-breaking interlayer Dzyaloshinskii–Moriya interactions in synthetic antiferromagnets. *Nat. Mater.* **18**, 679–684 (2019).
17. Zhang, H. *et al.* Room-temperature skyrmion lattice in a layered magnet (Fe0.5Co0.5)5GeTe2. *Sci. Adv.* **8**, eabm7103 (2022).
18. Bello, J.-L. *et al.* Field-free current-induced magnetization switching in GdFeCo: A competition between spin–orbit torques and Oersted fields. *J. Appl. Phys.* **132**, 083903 (2022).
19. Manchon, A. *et al.* Current-induced spin-orbit torques in ferromagnetic and antiferromagnetic systems. *Rev. Mod. Phys.* **91**, 035004 (2019).
20. Liu, L., Lee, O. J., Gudmundsen, T. J., Ralph, D. C. & Buhrman, R. A. Current-Induced Switching of Perpendicularly Magnetized Magnetic Layers Using Spin Torque from the Spin Hall Effect. *Phys. Rev. Lett.* **109**, 096602 (2012).
21. Hayashi, M., Kim, J., Yamanouchi, M. & Ohno, H. Quantitative characterization of the spin-orbit torque using harmonic Hall voltage measurements. *Phys. Rev. B* **89**, 144425 (2014).

22. Avci, C. O. *et al.* Interplay of spin-orbit torque and thermoelectric effects in ferromagnet/normal-metal bilayers. *Phys. Rev. B* **90**, 224427 (2014).
23. DC, M. *et al.* Room-temperature high spin–orbit torque due to quantum confinement in sputtered BixSe(1–x) films. *Nat. Mater.* **17**, 800–807 (2018).
24. Shao, Q. *et al.* Strong Rashba-Edelstein Effect-Induced Spin–Orbit Torques in Monolayer Transition Metal Dichalcogenide/Ferromagnet Bilayers. *Nano Lett.* **16**, 7514–7520 (2016).
25. Wu, H. *et al.* Room-Temperature Spin-Orbit Torque from Topological Surface States. *Phys. Rev. Lett.* **123**, 207205 (2019).
26. Pai, C.-F., Ou, Y., Vilela-Leão, L. H., Ralph, D. C. & Buhrman, R. A. Dependence of the efficiency of spin Hall torque on the transparency of Pt/ferromagnetic layer interfaces. *Phys. Rev. B* **92**, 064426 (2015).
27. Nguyen, M.-H., Ralph, D. C. & Buhrman, R. A. Spin Torque Study of the Spin Hall Conductivity and Spin Diffusion Length in Platinum Thin Films with Varying Resistivity. *Phys. Rev. Lett.* **116**, 126601 (2016).
28. Alghamdi, M. *et al.* Highly Efficient Spin-Orbit Torque and Switching of Layered Ferromagnet Fe3GeTe2. *Nano Lett.* **19**, 4400–4405 (2019).
29. Wang, X. *et al.* Current-driven magnetization switching in a van der Waals ferromagnet Fe3GeTe2. *Sci. Adv.* **5**, eaaw8904 (2019).
30. Zahnd, G. *et al.* Spin diffusion length and polarization of ferromagnetic metals measured by the spin-absorption technique in lateral spin valves. *Phys. Rev. B* **98**, 174414 (2018).
31. Bass, J. & Pratt, W. P. Spin-diffusion lengths in metals and alloys, and spin-flipping at metal/metal interfaces: an experimentalist's critical review. *J. Phys. Condens. Matter* **19**, 183201 (2007).
32. Kao, I.-H. *et al.* Deterministic switching of a perpendicularly polarized magnet using unconventional spin–orbit torques in WTe2. *Nat. Mater.* **21**, 1029–1034 (2022).
33. Shin, I. *et al.* Spin–Orbit Torque Switching in an All-Van der Waals Heterostructure. *Adv. Mater.* **34**, 2101730 (2022).
34. Ostwal, V., Shen, T. & Appenzeller, J. Efficient Spin-Orbit Torque Switching of the Semiconducting Van Der Waals Ferromagnet Cr2Ge2Te6. *Adv. Mater.* **32**, 1906021 (2020).
35. Gupta, V. *et al.* Manipulation of the van der Waals Magnet Cr2Ge2Te6by Spin-Orbit Torques. *Nano Lett.* **20**, 7482–7488 (2020).
36. Mogi, M. *et al.* Current-induced switching of proximity-induced ferromagnetic surface states in a topological insulator. *Nat. Commun.* **12**, 1404 (2021).
37. Li, W. *et al.* Room-Temperature van der Waals Ferromagnet Switching by Spin-Orbit Torques. *Adv. Mater.* **35**, 2303688 (2023).
38. Kajale, S. N., Nguyen, T., Hung, N. T., Li, M. & Sarkar, D. Field-free deterministic switching of all-van der Waals spin-orbit torque system above room temperature. *Sci. Adv* **10**, eadk8669 (2024).
39. Gweon, H. K. *et al.* Exchange Bias in Weakly Interlayer-Coupled van der Waals Magnet Fe3GeTe2. *Nano Lett.* **21**, 1672–1678 (2021).
40. Yi, C. *et al.* Ultrahigh Exchange Bias Field/Coercive Field Ratio in In Situ Formed Two-Dimensional Magnetic Te-Cr2O3/Cr5Te6 Heterostructures. *Adv. Mater.* **37**, 2410816 (2025).
41. Lu, L. *et al.* Tunable Magnetism in Atomically Thin Itinerant Antiferromagnet with Room-Temperature Ferromagnetic Order. *Nano Lett.* **24**, 5984–5992 (2024).
